# Aerobic exercise on the treadmill combined with transcranial direct current stimulation on the gait of people with Parkinson’s disease: A protocol for a randomized clinical trial

**DOI:** 10.1371/journal.pone.0300243

**Published:** 2024-04-25

**Authors:** Gabriel Antonio Gazziero Moraca, Diego Orcioli-Silva, Beatriz Regina Legutke, Pedro Paulo Gutierrez, Thiago Martins Sirico, Vinicius Cavassano Zampier, Victor Spiandor Beretta, Lilian Teresa Bucken Gobbi, Fabio Augusto Barbieri

**Affiliations:** 1 Posture and Gait Studies Laboratory, Department of Physical Education, Institute of Biosciences, São Paulo State University, Rio Claro, São Paulo, Brazil; 2 Human Movement Research Laboratory, Department of Physical Education, School of Sciences, São Paulo State University, Bauru, São Paulo, Brazil; 3 School of Technology and Sciences, Department of Physical Education, São Paulo State University, Presidente Prudente, São Paulo, Brazil; PLoS ONE, UNITED STATES

## Abstract

Gait impairments negatively affect the quality of life of people with Parkinson’s disease (PwPD). Aerobic exercise (AE) is an alternative to alleviate these impairments and its combination with transcranial direct current stimulation (tDCS) has demonstrated synergistic effects. However, the effect of multitarget tDCS application (i.e., motor, and prefrontal cortices simultaneously) combined with physical exercise on gait impairments is still little known. Thus, the proposed randomized clinical trial will verify the acute effects of AE combined with tDCS applied on motor and prefrontal cortices separately and simultaneously on gait (spatial-temporal and cortical activity parameters) in PwPD. Twenty-four PwPD in Hoehn & Yahr stages I-III will be recruited for this crossover study. PwPD will practice AE on treadmill simultaneously with the application of anodal tDCS during four intervention sessions on different days (∼ one week of interval). Active tDCS will be applied to the primary motor cortex, prefrontal cortex, and both areas simultaneously (multitarget), with an intensity of 2 mA for 20 min. For sham, the stimulation will remain at 2 mA for 10 s. The AE will last a total of 30 min, consisting of warm-up, main part (20 min with application of tDCS), and recovery. Exercise intensity will be controlled by heart rate. Spatial-temporal and cortical activity parameters will be acquired before and after each session during overground walking, walking with obstacle avoidance, and walking with a cognitive dual task at self-preferred velocity. An accelerometer will be positioned on the fifth lumbar vertebra to obtain the spatial-temporal parameters (i.e., step length, duration, velocity, and swing phase duration). Prefrontal cortex activity will be recorded from a portable functional near-infrared spectroscopy system and oxygenated and deoxygenated hemoglobin concentrations will be analyzed. Two-way ANOVAs with repeated measures for stimulation and moment will be performed. The findings of the study may contribute to improving gait in PwPD. Trial registration: Brazilian Clinical Trials Registry (RBR-738zkp7).

## Introduction

More than six million people are affected by Parkinson’s disease nowadays [1]. Parkinson’s disease is characterized by the progressive loss of dopaminergic neurons in the substantia nigra pars compacta of the basal ganglia [2, 3]. The lack of dopamine causes an imbalance in the inhibitory and excitatory signals sent via the thalamus from the basal ganglia to subcortical and cortical areas (such as the motor cortex). In short, there is an increase in inhibitory signals (GABAergic) from the basal ganglia to the thalamus, and consequently, the thalamus presents an underactive excitatory signal (glutamatergic activity) to the motor cortex, generating hypoactivation of this region [2, 3]. Because of this imbalance, people with Parkinson’s disease (PwPD) may experience rigidity, bradykinesia, resting tremors, postural instability, and impaired walking.

PwPD shows reduced gait velocity, stride length, and swing phase, besides the increase of double support time [4] and gait variability [5] during overground walking compared to neurologically healthy individuals, reducing gait automaticity [6]. Similar gait impairments were also reported during walking with obstacle avoidance [7–9] and with cognitive dual task in PwPD [10, 11]. In addition, changes in cortical activity, measured using functional near-infrared spectroscopy (fNIRS), during walking were reported in previous studies [11–14]. PwPD showed increased activity in the prefrontal cortex (PFC) during overground walking [13, 14], walking with obstacles [13, 15] and walking with cognitive dual task [11, 12] compared to neurologically healthy individuals. The increased recruitment of the prefrontal area can be considered a compensatory neural activity to deal with gait impairments and cortico-basal ganglia impaired pathways [16]. In addition to levodopa-based treatment, incorporating physical exercise can enhance the gait of PwPD.

Aerobic exercise (AE), particularly when performed on a treadmill, has shown promising results in enhancing gait parameters. For example, Pohl and colleagues [17] demonstrated improvements in gait velocity, stride length, and double support time after a single session of AE compared to traditional gait training. Multiple AE sessions have also shown positive effects on gait parameters in PwPD [18–20]. In addition to the behavioral effects of walking, treadmill exercise may increase corticomotor excitability in PwPD [21]. There is evidence that combining physical exercise, including AE, with transcranial direct current stimulation (tDCS) could have even more positive effects on spatial-temporal and cortical activity parameters during walking [22, 23].

tDCS has been suggested as a potential clinical tool to improve gait deficits in PwPD [24, 25]. Some authors have demonstrated benefits resulting from the isolated application of tDCS during walking [26–28], while other studies have reported no positive effects [29–32]. Furthermore, a recent meta-analysis indicated that applying tDCS alone does not lead to improvements in short-term motor symptoms of PwPD, including walking [33]. There is a body of evidence suggesting that combining tDCS with physical exercise can promote greater synergistic effects compared to interventions applied separately [34]. In a previous study, the combination of physical training focused on gait and balance with tDCS applied to the primary motor cortex (M1) resulted in a greater increase in gait velocity than either stimulation or exercise alone [22]. In contrast, a single session of AE on the treadmill improved gait velocity and stride length, but these positive effects were not enhanced by tDCS application over the M1 [35]. Indeed, Conceição and colleagues [23] applied AE on a stationary bike with tDCS over PFC and demonstrated a decrease in step time variability, along with an increase in PFC activity in the stimulated hemisphere during walking. This last result suggests that combining tDCS with AE may enhance the utilization of prefrontal resources in gait control, facilitating the compensatory mechanism (greater use of the indirect locomotor pathway) [16]. However, the combined effect of simultaneous stimulation of M1 and PFC with AE has not been tested yet. Stimulating both brain areas may be beneficial, as M1 is associated with the execution of voluntary movements [36] and is hypoactive in PwPD, while PFC acts as a compensatory mechanism for gait control [16].

Given the promising positive effects of stimulating the M1 and PFC during AE, this randomized clinical trial protocol aims to investigate the acute effects of tDCS applied to different target areas combined with AE on the treadmill on spatial-temporal and cortical activity parameters during overground walking, walking with obstacle avoidance and walking with cognitive dual-task in PwPD.

## Materials and method

### Study design and setting

**Fig 1:**
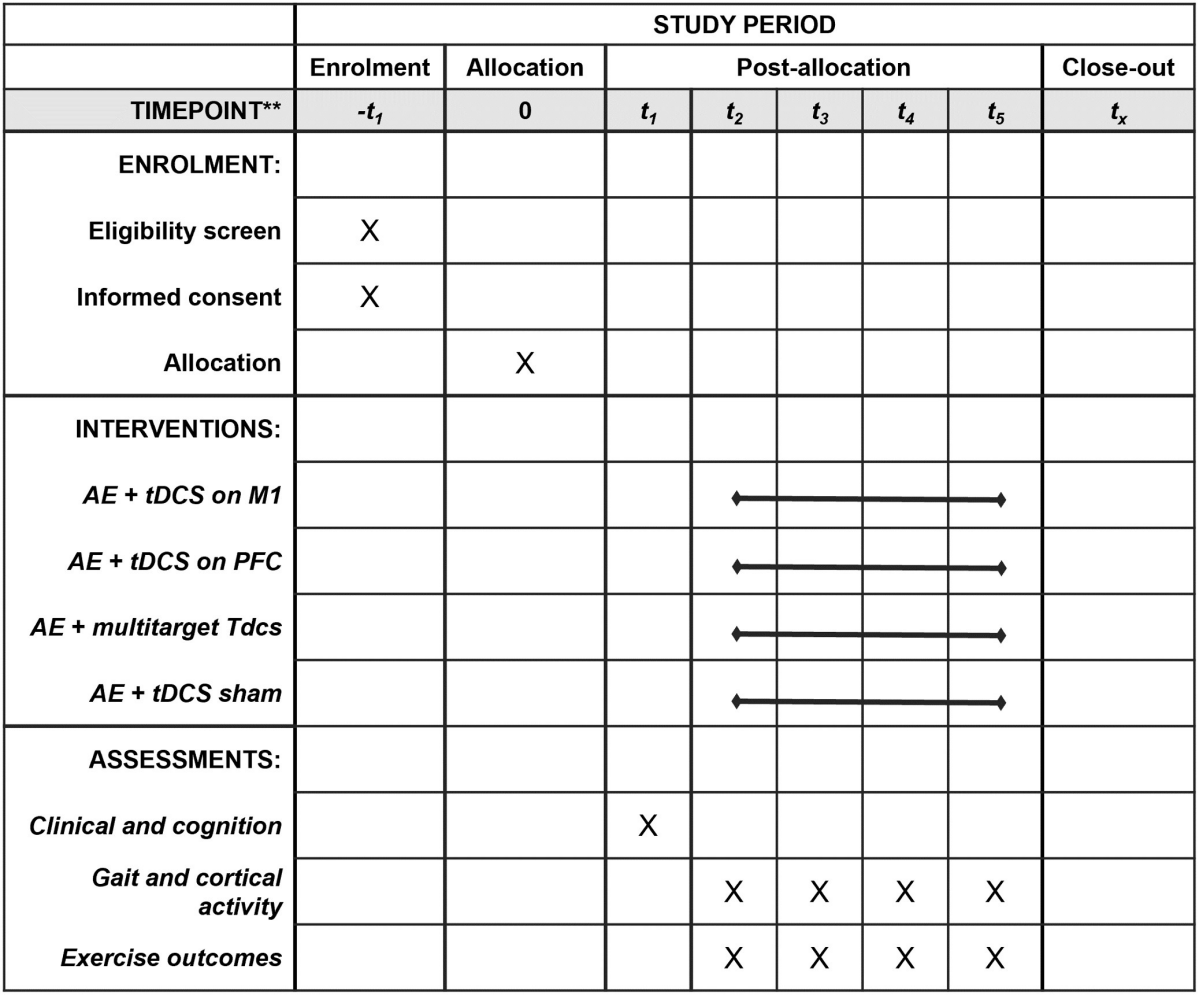
SPIRIT diagram of enrollment, interventions, and assessments.

A randomized, crossover, double-blind, sham-controlled clinical trial will be conducted at the Posture and Gait Studies Laboratory (LEPLO) of São Paulo State University (Unesp), Institute of Biosciences, Rio Claro. This study protocol adheres to the Standard Protocol Items: Recommendations for Interventional Trials (SPIRIT) checklist (S1 Checklist) [37]. The project was approved by the research ethics committee of the same University (CAAE: 54476421.1.0000.5465) and is registered on the Brazilian Clinical Trials Registry platform (RBR-738zkp7) with the Universal Trial Number (UTN) code U1111–1277-3248. Other documents on ethical appraisal can be found in the supporting information (S1–S4 Files). PwDP will visit the laboratory for non-consecutive five days, with screening assessments and treadmill familiarization conducted during the first visit. During the remaining four visits (minimum of one week apart), participants will undergo pre- and post-assessments (gait and cortical activity) and a session of AE combined with anodal tDCS (applied on M1, PFC, multitarget, or sham condition). All experimental procedures will be performed at the same time of the day for each PwPD and during the ON state of Parkinson’s disease medication. Fig 1 shows a SPIRIT diagram for the timeline of procedures.

### Participants

#### Sample recruitment and selection

This study will employ the convenience sampling technique, selecting, and recruiting PwPD from the community-dwelling. The sample size was calculated in G*Power software version 3.1, based on data from Fernandez-Lago colleagues [35]. The primary outcome and statistical tests used were gait velocity and Two-way ANOVAs with repeated measures (within factors), respectively (alpha = 0.05; beta = 0.08; ηp^2^ = 0.43). The sample size analysis indicates a minimum of 16 PwPD is required for this study. Considering the potential for sample loss, we will invite 24 PwPD to participate in the clinical trial through personal and/or telephone contact (from November 10^th^, 2023, to March 31^st^, 2024). In case we have a positive reply, a member of the research team will explain the study’s objectives, risks, benefits, and ethical implications. If participants consent to participate in the study, they will be asked to sign two copies of an informed consent form (one for themselves and the other for the member of the research team).

#### Inclusion criteria

We will include only individuals who have received a clinical diagnosis of idiopathic Parkinson’s disease from a board-certified neurologist, in accordance with the UK Brain Bank criteria [38], and who have independent locomotion.

#### Exclusion criteria

We will exclude individuals who meet any of the following criteria: (i) have another neurological disease in addition to Parkinson’s disease; (ii) be above stage III of the adapted Hoehn & Yahr (H&Y) scale [39]; (iii) have characteristics that make the tDCS application unsafe, such as metallic skull implant or epilepsy; (iv) any orthopedic and/or vision problems that impossibilities to comply with the experimental protocol and; (v) cognitive decline (score below 24 points on the Mini-Mental State Examination—MMSE) [40]. PwPD who change their medications during the study will also be excluded.

#### Confidentiality

Each participant will be assigned an identification code to ensure anonymity throughout the research. Information that could potentially identify participants will not be disclosed at any point. Access to the collected, processed, and analyzed data will be restricted to the responsible researcher and his team exclusively. The data will be utilized solely for academic research purposes, and dissemination will take place through conferences and scientific articles.

### Strategies to increase intervention adherence

Assessors will maintain weekly communication with participants, either through telephone contact or in person, to gather feedback and address any potential discomfort related to the intervention. Furthermore, interventions will be scheduled based on the participants’ availability to ensure their engagement and maintain continuity throughout the intervention.

### Randomization and blinding

The order of intervention sessions will be randomized and counterbalanced across participants in a 1:1:1:1 ratio using an online random number generator (www.randomization.com). A member of the research team, not involved in the experimental procedures, will conduct the randomization, and maintain allocation confidentiality until the session begins. Two strategies will be implemented to ensure study blinding: i) participants will be unaware of the type of stimulation applied in each session and ii) the team member conducting the AE and tDCS sessions will not participate in the pre-and post-assessments.

### Assessments

#### Clinical and cognition

The motor severity of Parkinson’s disease, the stage of disease, and cognitive screening will be assessed by the Movement Disorders Society—Unified Parkinson’s Disease Rating Scale motor part (MDS-UPDRS III) [41], the adapted H&Y scale [39], and the MMSE [40], respectively. An experienced and trained evaluator will perform these assessments. The fear of falls and freezing of gait will be assessed by the Falls Efficacy Scale—International [42] and Freezing of Gait Questionnaire [43], respectively.

#### Gait and cortical activity while walking

Initially, trained evaluators will mark the Cz and Fpz positions on the participant’s head, corresponding to the 10–20 electroencephalography (EEG) system. The Cz position will be determined as the midpoint between the nasion and inion, and the pre-auricular points. The Fpz position will be marked approximately 13.5 cm below Cz, towards the forehead. These markings will serve as a guide for positioning a Neoprene cap, equipped with fNIRS optodes, on the participant’s head to record PFC activity during walking. The fNIRS optodes will be placed on the frontal part of the head, corresponding to Brodmann areas 9, 10, and 46 of both the left and right hemispheres, representing the dorsolateral and anterior PFC [15, 44, 45]. A tri-axial accelerometer will be positioned over the fifth lumbar vertebra of the participant to obtain gait parameters. The lumbar height will be individualized for each participant, recorded, and replicated in all assessments. After the preparations, participants will walk on a 26.8 m long circuit at a normal and comfortable pace for three experimental conditions: i) usual walking; ii) obstacle avoidance, and iii) with a cognitive dual task. Three trials will be conducted for each condition randomly, and the order of conditions will be identical in pre- and post-assessments. Each trial’s total duration will be 60 s (30 s standing and 30 s walking). During the standing still period (baseline), participants will be instructed to stand quietly, look straight ahead, and perform simple mental counting (addition of one by one). This task aims to standardize attentional demand during the baseline [46]. After a verbal signal (“ready, go”), the participant will perform the experimental task for 30 s. In the obstacle avoidance condition, four foam obstacles (60 cm long x 5 cm wide x 15 cm high) will be arranged along the circuit at a uniform distance between them [15, 45]. In the cognitive dual-task condition, an audio containing random numbers from 1 to 9 will play during the 30 s of walking. The participant must mentally count how many times a certain class of numbers (even or odd) was expressed [11, 45]. At the end of the pre-assessment, the fNIRS cap will be removed to carry out the intervention protocol, but appointments will be made to ensure the same positioning in the post-intervention assessment.

### Protocol of aerobic exercise combined with tDCS

**Fig 2:**
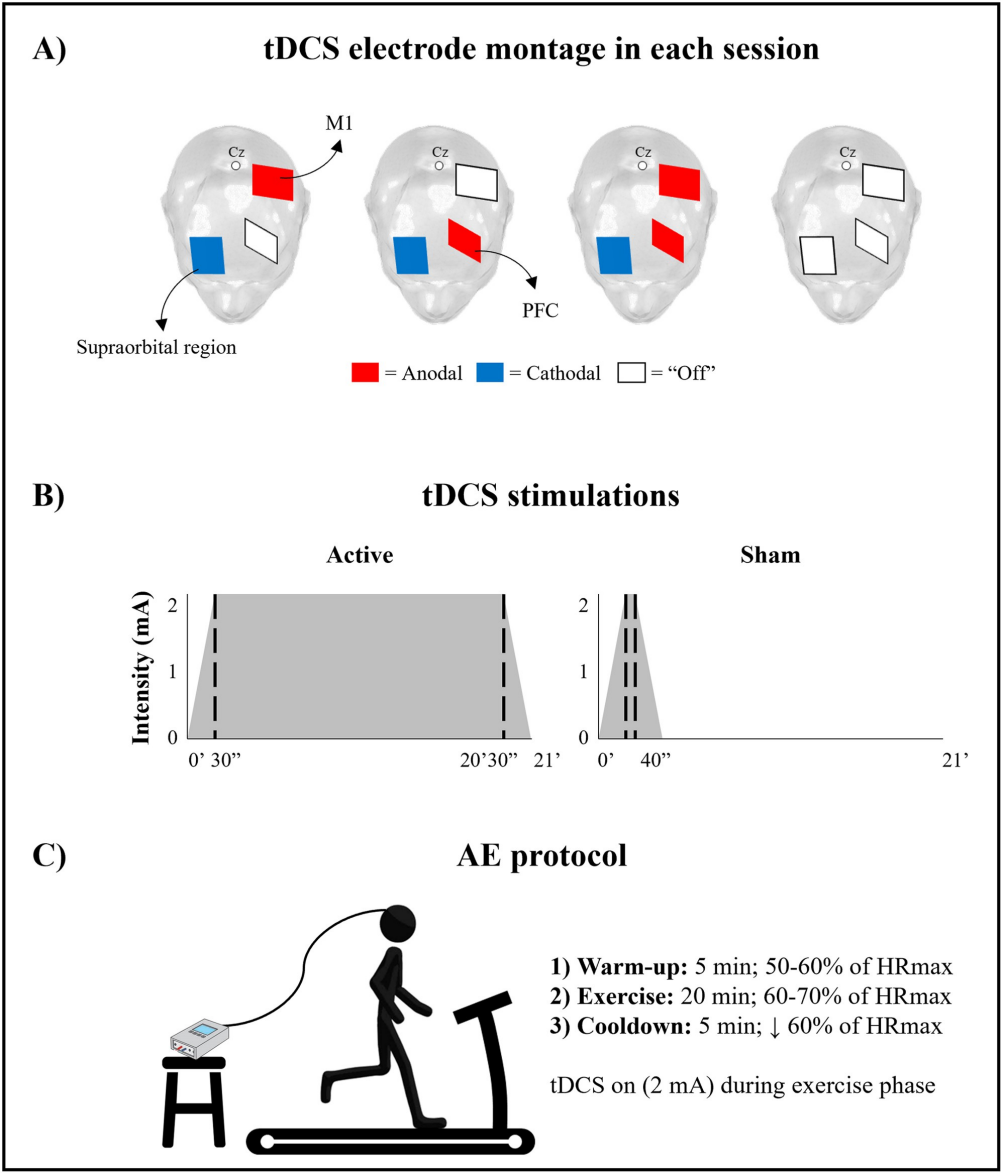
Experimental setup. A: tDCS electrode montages with three electrodes in all sessions. B: tDCS protocols for active and sham sessions. C: Protocol of aerobic exercise on treadmill.

Participants will be seated on a comfortable chair for tDCS preparations, blood pressure measurement, and resting heart rate. tDCS will be delivered by a MicroEstim Genius and/or MicroEstim Foco Research stimulator (NKL Electronic Products Ltda.—EEP, Brusque/Santa Catarina, Brazil) through conductive-rubber electrodes, placed in saline-soaked sponges (35 cm^2^, current density = 0.057 mA/cm^2^). The stimulation will target different regions in each session: active tDCS on M1, PFC, or multitarget, and a sham condition (Fig 2A). For multitarget, tDCS, two stimulators will be used, and the cathodal cable of the equipment will be linked to an inverted Y splice (NKL Electronic Products Ltda.—EEP, Brusque/Santa Catarina, Brazil) connected to the cathode electrode. In all sessions, anodal electrodes will be positioned according to the 10–20 EEG system, on M1 (C3/C4) and PFC (F3/F4), while the cathodal electrode will be positioned over the contralateral supraorbital region (FP1/FP2). Anodal stimulations will be applied to the cerebral hemisphere contralateral to the body side most affected by Parkinson’s disease, determined by MDS-UPDRS III items (3.3b-3.17d). The placement of three electrodes in all sessions will enhance participant blinding, making it more challenging to deduce the region and type (active or sham) of the stimulation being performed. Active tDCS stimulations (2 mA) will be delivered for 20 min, with a 30-seconds ramp-up at the beginning and a 30-seconds ramp-down at the end of the stimulation period (Fig 2B). For sham stimulation, the same 30-seconds of ramp-up and ramp-down will be applied, but the active stimulation will last only 10 s (Fig 2B). The stimulator will remain off for the next 19 min and 50 s.

AE will be conducted on a Millenium ATL treadmill (Inbramed—Brazilian Medical Equipment Industry Ltda., Porto Alegre/Rio Grande do Sul, Brazil). The intensity of exercise will be personalized for each participant and controlled by the percentage of their maximum heart rate (HRmax). Continuous monitoring and recording of the heart rate will be carried out using the Polar V800 (Polar Electro Brazil Commerce, Distribution, Importation, and Exportation Ltda., São Paulo/SP, Brazil). HRmax of each participant will be estimated using Eq 1, which has been previously validated [47]. The Eq 2 will be applied specifically for participants using β-blocker medication [48].

HRmax=208-(Age×0.7) (1)

HRmax=164-(Age×0.7) (2)

The exercise protocol will last 30 min and will consist of three parts: i) 5-minute warm-up phase (maintaining the HR between 50-60% of HRmax); ii) 20-minute exercise phase (maintaining HR between 60-70% of HRmax); and iii) 5-minute cooldown phase (maintaining HR below 50% of HRmax). tDCS will be applied during the 20-minute exercise phase and will be off in warm-up and cooldown phases (Fig 2C). The evaluator will increase and/or decrease the treadmill velocity to ensure that the percentage of HRmax remains within the specified target limits. The subjective perception of exertion (SPE) will be collected using the modified Borg scale (0–10 points) [49]. SPE will be recorded immediately before the exercise begins, every 5 min, and immediately after the end of the exercise. Additionally, other intervention outcomes will be recorded, such as treadmill velocity every 5 min and the total distance covered by the participant. For safety, all participants will be allowed to hold on to the handrails. A structured questionnaire will be administered immediately after the session to evaluate the potential side effects of tDCS [50, 51], and blood pressure will be measured again. At the end of the last intervention session, the evaluator will inquire with the participant about which session they received the sham condition. This approach will be implemented due to the extensive minimum interval between the first and last sessions (one month), making it challenging for participants to recall the sensations or discomforts experienced during each tDCS session.

### Data analysis and processing

A tri-axial accelerometer (Trigno^™^ Wireless System, Delsys, Inc., Natick Massachusetts, USA), sampling at 148.15 Hz, will record the center of mass acceleration during gait assessments. EMGWorks^®^ 4.7.5 (Delsys, Inc., Natick, Massachusetts, USA) will be used for data collection. Gait parameters will be derived from vertical acceleration data algorithms previously validated [52, 53] in the Matlab^®^ R2022b software (The MathWorks, Inc., Natick, Massachusetts, USA). In summary, the acceleration data will be transformed using a vertical-horizontal coordinate system and filtered with a 4th-order Butterworth filter, having a cutoff frequency of 20 Hz [54, 55]. The initial and final contacts of the gait cycle will be estimated using the continuous wavelet transform (CWT) of vertical acceleration. The initial and final contact events will be identified through the minimum and maximum points of the CWT, respectively [52]. Step velocity, step time, step length, and swing phase will be calculated. Additionally, the variability of each gait parameter will be calculated by the coefficient of variation.

PFC activity will be registered using a mobile 8-channel fNIRS device (OctaMon Artinis Medical Systems, Elst, The Netherlands), with a collection frequency of 10 Hz. The Octamol model is equipped with eight light transmitters and two light detectors (four transmitters and one detector per hemisphere), that detect changes in concentrations of oxygenated (HbO_2_) and deoxygenated (HHb) hemoglobin. This system employs infrared light with wavelengths of 760 and 850 nm and an interoptode distance of 35 mm. The OctaMon model utilizes a Bluetooth connection, enabling participants to walk without the inconvenience of wires. OxySoft 3.0.52 software (Artinis Medical Systems, Elst, The Netherlands) will be used for data collection, storage and calculation of HbO_2_ and HHb concentrations. The modified Beer-Lambert law will be applied to convert the optical density of the raw signal into HbO_2_ and HHb concentrations. The differential path factor will be set to 6.61 for all participants.

The fNIRS signal analysis procedures (artifact corrections and filtering) will be carried out using the open-source software NIRS-SPM (http://www.fil.ion.ucl.ac.uk/spm/software/) [56] following previous recommendations [57]. In summary, a low-pass filter based on the canonical hemodynamic response function will be used to reduce high-frequency noise. Additionally, a wavelet algorithm will decompose the signal into global trends, hemodynamic responses, and movement artifacts. Preprocessed data will be exported to Matlab^®^ R2022b software, and a customized algorithm will be used for additional processing. HbO_2_ and HHb concentrations will be analyzed for each hemisphere separately (interpreted by stimulated and non-stimulated). The means of these parameters will be calculated considering four channels per hemisphere. Subsequently, data analysis will be divided into baseline and task periods. The baseline period will consist of 10 s before starting walking, and the task period will be from the 5th to the 25th second after starting walking. The mean concentrations of HbO_2_ and HHb will be calculated for each period, and the differences between periods for each parameter will be utilized to evaluate the relative change in cortical activity.

### Statistical analysis

Statistical analysis will be conducted using SPSS 25.0 software (International Business Machines Corporation, Armonk, New York, USA), with a significance level set at *p* < 0.05. Clinical, cognitive, and demographic characteristics will be summarized using means and standard deviations or medians and quartiles (25–75) based on the normality of data distribution. The side effects of tDCS will be analyzed with Friedman’s ANOVA. Means and standard deviations will be calculated for primary, second, and intervention outcomes (treadmill velocity and total distance covered, SPE, and percentage of HRmax). Two-way ANOVA with repeated measures for stimulation (M1 x multitarget x PFC x sham) and moment (pre and post) will be employed to analyze gait parameters and activity cortical in each walking condition separately (usual, obstacle avoidance, and dual task). The Mauchly test will be used to check sphericity, and the Greenhouse-Geisser correction will be adopted if necessary. In the case of interaction between factors, Bonferroni post hoc tests with adjusted significance levels will be utilized to identify differences. Finally, for each combination of AE and tDCS, the Standardized Response Mean will be calculated as the effect size of the intervention, considering the mean difference between moments (post—pre) divided by the standard deviation of change.

## Discussion

This study outlines a randomized clinical trial protocol designed to examine the acute effects of AE on a treadmill combined with tDCS on spatial-temporal gait parameters and PFC activity in PwPD. Our protocol includes gaits assessments in various contexts, including usual walking, walking with obstacles, and performing a cognitive task while walking. This comprehensive approach allows us to gain a better understanding of the immediate effects of the combined intervention on the gait of PwPD. Additionally, we will compare the effects of different tDCS setups in combination with physical exercise, with a particular emphasis on multitarget stimulation.

The most innovative feature of the proposed intervention is multitarget tDCS. This study marks the first attempt to verify the effects of simultaneously applying tDCS to both M1 and PFC, in conjunction with physical exercise for PwPD. M1 plays a crucial role in carrying out movements associated with walking [36] and serves as the primary contributor to the direct locomotor pathway. PFC contributes to the indirect locomotor pathway by reallocating cognitive resources to improve gait control in populations with impaired direct locomotor pathway, such as PwPD [16]. Stimulation of M1 may facilitate movement execution, while stimulation of the PFC may augment cognitive resources to improve gait automaticity of PwPD. Therefore, simultaneously stimulating both the M1 and PFC may prove beneficial to the gait of this population. Two prior studies explored the effects of multitarget tDCS applied alone [58, 59]. In a pilot study conducted by Dagan and colleagues [58] it was verified that multitarget tDCS reduced the severity of freezing of gait after a single session, compared to stimulation focused solely on M1. Encouraged by these initial findings, the researchers initiated a larger study with a substantial sample size (71 PwPD) and 15 multitarget tDCS sessions (10 intervention and 5 maintenance) [59]. However, multitarget tDCS did not yield significant effects in the FOG-provoking test [59]. More studies are needed to try to understand the effects of multitarget tDCS on the gait of PwPD It is noteworthy that both studies employed high definition tDCS, a method using circular electrodes much smaller than conventional tDCS electrodes. This design allows for a more precise direction of the electric current to the target areas. However, conventional tDCS is low-cost, and studies testing its effects are necessary for the future use of this tool in clinical practice for PwPD.

Setting an appropriate AE intensity on the treadmill is crucial to guarantee reliable results. We chose to control the intensity by heart rate, using recommended limits for the practice of AE in PwPD [60]. We expect to find a treadmill velocity that allows the individual to walk within the limits and this velocity will be maintained in all training sessions. However, previous studies demonstrated promising results using increments in treadmill velocity within each session [18, 61]. For example, Cakit and colleagues [18] applied increments of 0.6 km/h every 5 min, until the participants reached their tolerated velocity on the treadmill. After eight weeks, PwPD improved gait, assessed by Dynamic Gait Index, and measures of balance and fear of falls [18]. Another study compared treadmill training with and without visual and auditory cues, in which treadmill velocity was increased every three days (increments of 0.05 stride cycles/s) [61]. The authors observed improvements in gait velocity, stride length, walking capacity, and freezing of gait in both protocols after 28 consecutive sessions, but the positive effects were more pronounced in treadmill training with cues. Nevertheless, in the context of acute exercise, it appears that there is no difference between employing speed increments or maintaining a constant speed during the intervention session [17].

In this protocol, we aim to evaluate various locomotor tasks commonly encountered in individuals’ daily lives. This study seeks to broaden understanding of the effects of combining tDCS and AE on the gait of PwPD. Also, the protocol highlights the utilization of accessible and non-invasive portable tools for assessing and intervening in gait and cortical activity during walking, which is a notable strength. A single accelerometer will be employed to estimate gait parameters, a portable fNIRS system will estimate PFC activity during walking based on the hemodynamic response in the area, and tDCS will be used to maximize gains in gait. These equipment enhance the ecological validity of the protocol, bridging the gap between laboratory evaluations and clinical practice.

This clinical trial proposes a potential treatment for gait impairments in PwPD. A systematic has demonstrated that AE on a treadmill improves spatial-temporal gait parameters, such as walking velocity and stride length [62]. Moreover, combining tDCS with exercise can amplify the effects of AE, further enhancing the gait of PwPD. This integrated approach could serve as an efficient and accessible rehabilitation strategy in clinical practice.

## Supporting information

S1 Checklist SPIRIT checklist. (PDF)

S1 File Project approved by the ethics committee—English. (PDF)

S2 File Project approved by the ethics committee—Original. (PDF)

S3 File Informed consent for participants—English. (PDF)

S4 File Informed consent for participants—Original. (PDF)
