# Supplementary material for: Aerobic exercise on the treadmill combined with transcranial direct current stimulation on the gait of people with Parkinson’s disease: A protocol for a randomized clinical trial
Source: PLoS One. 2024 Apr 25;19(4):e0300243. doi: 10.1371/journal.pone.0300243 (PMC11045059; doi:10.1371/journal.pone.0300243)
Supplement: S1 File — (PDF) [file pone.0300243.s002.pdf]

EFFECTS OF TRANSCRANIAL DIRECT CURRENT STIMULATION COMBINED WITH TREADMILL TRAINING ON GAIT OF PEOPLE WITH PARKINSON'S DISEASE: A RANDOMIZED CLINICAL TRIAL

Proponent: Gabriel Antonio Gazziero Moraca

Research team: Profa. Dra. Lilian Teresa Bucken Gobbi; Prof. Dr. Diego Orcioli-Silva; Prof. Me. Victor Spiandor Beretta and Beatriz Regina Legutke.

Institution: São Paulo State University (Unesp), Rio Claro, Brazil.

ABSTRACT

Gait impairments affects the quality of life of people with Parkinson's disease (PD). Transcranial direct current stimulation (tDCS) has shown inconclusive results on walking, but evidence suggests that combining tDCS with physical exercise may be beneficial on gait of people with PD. The objective of this study is to verify the acute effects of anodal tDCS applied to the primary motor cortex (M1) and prefrontal cortex (PFC) combined with treadmill training on gait of people with PD. This study will be a randomized double-blind sham-controlled crossover trial. Ten PD people will be recruited to participate in this study for five days. On day 1, people will perform clinical and cognitive assessments, and treadmill familiarization (observation of comfortable treadmill speed). On days 2, 3, 4 and 5, people will participate in pre- and post-intervention assessments (walking and cortical activity), and combined intervention of anodal tDCS with treadmill training. The interval between intervention days will be one week and the sequence of exposure to sessions will be randomized between people. Active tDCS will be applied to the M1 and PFC (separately and simultaneously) and there will be a sham/placebo tDCS session. The treadmill training will consist of: i) 5 min warm-up; ii) main part of 20 min and; iii) 5 min cool down. For safety, people will be wearing a harness during the entire training session. The intensity of the training will be controlled by each patient's maximum heart rate. During treadmill training, people will receive active tDCS for 20 min or sham tDCS for 10 s (off in subsequent minutes). Before and after the intervention sessions, people will walk a 26.8m long circuit at their preferred speed under the following conditions: i) usual; ii) overcoming obstacles and; iii) with dual cognitive task. There will be 3 attempts in each condition and an accelerometer positioned on the fifth lumbar vertebra will be used to obtain gait parameters. Step speed and length, duration of the swing phase and double support and their respective variability will be analyzed. Prefrontal cortex activity during walking will be recorded using functional near-infrared light spectroscopy and the

oxyhemoglobin (HbO2) concentrations in both hemispheres will be analyzed. The deltas of the gait parameters and HbO2 concentration for each intervention session will be calculated. Two-way ANOVAs with repeated measures for the walking condition factors (usual X overcoming obstacle X cognitive dual task) and the stimulation condition factors (M1 X PFC X M1+PFC X sham) will be used. Bonferroni post hoc tests will be applied to identify differences. The significance level will be maintained at < 0.05 and the standardized response mean will be used to verify the magnitude of the intervention on the gait parameters.

Keywords: Cortical stimulation. Acute exercise. Locomotion. Adaptive walking. Dual task. Neurodegenerative diseases.

1 CONTEXT AND PROJECT JUSTIFICATION

Transcranial direct current stimulation (tDCS) is a technique that has been shown to be efficient in improving the motor symptoms of Parkinson's disease (PD), such as: bradykinesia (BENNINGER et al., 2010) and rigidity (FREGNI et al., 2006). However, studies regarding the effects of tDCS on gait of people with PD are still inconclusive. For example, a recent study showed a decrease in cadence, but did not find an increase in speed and stride length after anodal tDCS in the primary motor cortex (M1) and the supplementary motor area (DA SILVA et al., 2018). In contrast, Mishra and colleagues (2021) found an increase in walking speed after anodal tDCS applied to the prefrontal cortex (PFC). A possible explanation for these controversial results is the application of different tDCS protocols (intensity, duration, target region, etc.). Furthermore, other studies have combined tDCS with different motor interventions to improve gait of people with PD (KASKI et al., 2014a, 2014b; YOTNUENGNIT et al., 2018). However, results remain inconsistent. It is worth noting that applying tDCS with concomitant intervention (motor and/or cognitive) appears to be a promising strategy to improve the motor deficits generated by PD (BERETTA et al., 2020). Therefore, advancing the knowledge about the possible benefits of tDCS combined with a motor intervention on gait in people with PD is necessary.

Our research group, the Posture and Gait Studies Laboratory (LEPLO), has been dedicated to investigating the effects of tDCS with or without concurrent motor intervention on motor deficits in people with PD. In this sense, a recent study showed that anodal tDCS applied to the PFC combined with cycle ergometer exercise improves reaction time and cortical activity during walking, but not improves the gait parameters (CONCEIÇÃO et al., 2021). This study had limitations, such as stimulating only one area (PFC) and using the cycle ergometer. A recent meta-analysis suggests that stimulating more than one cortical area (at the same time or not) is more beneficial for walking control compared to stimulating just one area (LEE et al., 2019).

Furthermore, treadmill training can bring more significant benefits by encouraging motor gestures similar to walking. The present proposal seeks to advance the knowledge of the effects of this technique, applying it in more than one cortical region (PFC and M1) concomitantly with treadmill training, in walking with different difficulties (e.g., usual walking, dual-task walking, and walking overcoming obstacles). It is worth mentioning that treadmill training generates benefits in walking (MEHRHOLZ et al., 2015) and that the combination of this form of intervention with tDCS has been little explored in people with PD to date.

This proposal has scientific, technological, health and educational impacts. Regarding scientific impact, this proposal aims to advance the knowledge about the effects of tDCS combined with aerobic physical exercise on a treadmill on gait of people with PD. The results of this proposal will be disseminated in the following ways: oral communications and/or posters at scientific events in the areas of Motor Behavior and Neuroscience and articles in journals with an impact factor greater than 3 (e.g, *Neurorehabilitation & Neural Repair and/ or Parkinsonism & Related Disorders*). The technical competence that will be developed by the research members, in the use of portable equipment that evaluates walking control in "real time" in a biomechanical (accelerometer) and cortical (functional near-infrared light spectroscopy – fNIRS) manner, added to the application of non-invasive brain stimulation (tDCS), represent the technological impact of the proposal. As to the impact on health and also related to Sustainable Development Goal No. 3 of the United Nations, the findings resulting from this proposal will contribute to the optimization of intervention programs focused on improving walking in people with PD. The educational impact refers to the development of the candidate as a researcher and to the other members of LEPLO regarding the development of skills in collecting, analyzing data and discussing cortical activity, brain stimulation and physical exercise, and transferring the knowledge produced to the activities of undergraduate and postgraduate education. Therefore, the present proposal, in addition to contributing to the

understanding of the effects of tDCS combined with exercise on gait of people with PD, also represents gains in the candidate's training, in the research carried out at LEPLO and in the health of the investigated population.

2 INTRODUCTION

PD is the second most prevalent neurodegenerative disease in the world (ASCHERIO; SCHWARZSCHILD, 2016), with the number of cases more than doubling in the last 30 years (2.5 million people in 1990 versus 6.1 million individuals in 2016) (GBD 2016 PARKINSON’S DISEASE COLLABORATORS, 2018). Specifically in Brazil, PD affects around 3.3% of individuals over 64 years of age (BARBOSA et al., 2006). The pathophysiological characteristic of PD is the degeneration of dopaminergic neurons in the substantia nigra pars Compacta, responsible for the production of dopamine (GALVAN; WICHMANN, 2008; WICHMANN; DELONG, 2007). The loss of dopamine causes an imbalance in the inhibitory and excitatory signals that are sent by the basal ganglia to the motor cortex (PETERSON; HORAK, 2016; TAKAKUSAKI; TOMITA; YANO, 2008). As a consequence of this imbalance, people with PD present the following motor impairments: muscle stiffness (rigidity), tremor, bradykinesia (slowness of movement), hypometria (reduction in range of movement), postural instability and impaired walking (GALVAN; WICHMANN, 2008).

The gait of people with PD is characterized by decreased walking speed, step length and swing phase time, and increased time in double support as well as gait variability (MIRELMAN et al., 2019). Furthermore, people' walking deficits are exacerbated in more complex situations, such as walking while overcoming obstacles (GALNA; MURPHY; MORRIS, 2010; VITÓRIO et al., 2010, 2014) or walking while simultaneously performing a cognitive task (dual task) (KELLY; EUSTERBROCK; SHUMWAY-COOK, 2012; ORCIOLI-SILVA et al., 2020). Recently, studies have identified changes in cortical levels during walking in people with PD.

For example, studies using fNIRS observed that people with PD have greater PFC activity during usual walking compared to healthy older people (MAIDAN et al., 2016; STUART et al., 2019). Furthermore, PFC activity is increased during walking when overcoming obstacles (MAIDAN et al., 2016; ORCIOLI-SILVA et al., 2021b) and with dual task (NIEUWHOF et al., 2016; ORCIOLI-SILVA et al., 2020) compared to usual walking in people with PD. This increase in cortical activity during walking indicates that people use compensatory mechanisms to deal with walking impairments (HEROLD et al., 2017). Therefore, investigating forms of intervention that improve walking in various conditions present in people' daily lives is clinically important.

The most common treatment for the symptoms of PD is pharmacological, through the use of levodopa – a precursor of dopamine (NONNEKES et al., 2016; TARAZI et al., 2014). Pharmacological treatment improves some motor symptoms of PD, such as tremor and rigidity (NONNEKES et al., 2016), and improves walking speed and stride length (CURTZE et al., 2015; ORCIOLI-SILVA et al., 2020). Also, pharmacological treatment facilitates the recruitment of the PFC during walking overcoming obstacles (ORCIOLI-SILVA et al., 2021b) and with dual task (ORCIOLI-SILVA et al., 2020). However, prolonged use of the medication causes motor fluctuations, dyskinesias (involuntary movements) (JANKOVIC, 2000; NONNEKES et al., 2016) and some gait parameters are not responsive to the medication, such as the swing phase and gait variability (CURTZE et al., 2015; ROCHESTER et al., 2011). In this way, investigating the use of different interventions for the rehabilitation of people with PD, such as tDCS, is necessary.

The tDCS is a non-invasive brain stimulation technique that involves the application of low-intensity electrical currents to regions of interest in the cerebral cortex, capable of modulating cortical excitability, but insufficient to generate action potentials (NITSCHE et al., 2002, 2008). A direct current device delivers electrical currents (usually 1-2 mA) through anode

(positive) and cathode (negative) electrodes placed at specific locations on the scalp (BRUNONI et al., 2012; NITSCHE et al., 2008). Anodal stimulation causes an increase in excitability, while cathodal stimulation decreases the excitability of target areas (NITSCHE et al., 2002) Among brain stimulation techniques, tDCS stands out for its safety and ease of use, for the viability and low cost when compared to other brain stimulation techniques, such as transcranial magnetic stimulation. Although tDCS presents promising results, its effects on gait of people with PD are still controversial.

A recent meta-analysis demonstrated that tDCS generates a short-term beneficial effect on gait of people with PD, but the magnitude of this effect was small (LEE et al., 2019). Some studies have demonstrated improvement in walking after tDCS (DA SILVA et al., 2018; DAGAN et al., 2018; MISHRA; THRASHER, 2021), however, others have not (BENNINGER et al., 2010; BUENO et al., 2019; DORUK et al., 2014). Possible explanations for these findings are the different tDCS protocols applied. Furthermore, the application of tDCS with different concurrent (motor) interventions to improve walking (CONCEIÇÃO et al., 2021; YOTNUENGNIT et al., 2018) and the other motor symptoms of PD (BERETTA et al., 2020) is increasing in the literature. In this sense, a recent systematic review showed that combining tDCS with other forms of intervention seems to cause more benefits in walking than applying tDCS alone (POL et al., 2021). For example, combining anodal tDCS in M1 with physical training improved walking speed, but this effect was not found with the application of tDCS alone (KASKI et al., 2014b). Furthermore, combining tango sessions with anodal tDCS applied to M1 was able to improve walking endurance (6-minute test) (KASKI et al., 2014a). Stimulating M1 is justified by the fact that this area is involved in the execution of movements related to walking (PETERSEN et al., 2012). Another study combined anodal tDCS applied to the PFC with cycle ergometer training and found improvement in the swing phase, but did not verify improvement in several other gait parameters (walking speed, double support time, step

length, etc.) (CONCEIÇÃO et al., 2021). The PFC is capable of allocating attentional resources during walking and is involved in the indirect locomotor pathway, which is more activated in individuals with impaired direct locomotor pathways, such as people with PD (HEROLD et al., 2017). Finally, other studies found similar results between tDCS applications alone and combined with specific walking training (COSTA-RIBEIRO et al., 2017) and physiotherapy (YOTNUENGNIT et al., 2018). Performing tDCS with physical training seems to be a promising strategy to improve the walking of people with PD, but there is still no consensus on what the ideal cortical area is to be stimulated and what is the best concurrent intervention.

Based on the above, the general objective of this study is to verify the acute effects of anodal tDCS applied to the M1 and PFC combined with treadmill training on gait of people with PD. The specific objective is to compare the effects between the combination of tDCS applied individually in the M1 and PFC with treadmill training and the combination of tDCS applied simultaneously in these regions with treadmill training on gait parameters. The secondary objective is to analyze the influence of combining tDCS with treadmill training on PFC activation in different walking conditions, such as usual walking, overcoming obstacles and dual task. Since studies have shown benefits of tDCS (LEE et al., 2019) and treadmill training (MEHRHOLZ et al., 2015) on people’ gait, we expect that combining tDCS with treadmill training will enhance improvements in speed and variability of walking and that increases PFC activation during walking in different conditions.

3 METHODS

3.1 Participants

The convenience sampling method will be used, and people with PD will be recruited and selected from the database of the Physical Activity Program for People with Parkinson's disease (PROPARKI). A priori analysis in the G*Power 3.1 software was carried out based on a

previous study by our group (CONCEIÇÃO et al., 2021) by inserting the following parameters: partial eta-squared = 0.27; statistical power = 0.80 and $\alpha < 0.05$. The variable and statistical test used in the calculation were walking speed and two-way ANOVA for repeated measures (within factors), respectively. The analysis indicated a total sample size of 28 participants; however, 7 participants would already be enough due to the project having a crossover design. In order to prevent sample losses, 10 people will be invited to participate in the research. As an inclusion criterion, people must have a diagnosis of idiopathic PD given by a private neurologist, according to the UK Parkinson's Disease Society Brain Bank criteria (HUGHES et al., 1992). The exclusion criteria will be: i) having another neurological disease in addition to PD; ii) be above stage 3 on the adapted Hoehn & Yahr (H&Y) scale (GOETZ et al., 2004); iii) have characteristics that make tDCS risky (neural implants; history of seizures; pacemaker; epilepsy); iv) history of orthopedic and/or vision problems that make it impossible to participate in the experimental procedures and v) indicative of dementia (score <20 on the Mini-Mental State Examination – MMSE) (BRUCKI et al., 2003).

3.2 Experimental design

This research project will be a randomized double-blind sham-controlled crossover trial and will follow the recommendations of the CONSORT checklist (Appendix A). All experimental procedures will be carried out on the premises of LEPLO, at the São Paulo State University (Unesp), Institute of Biosciences, Rio Claro. The project will be submitted to the Research Ethics Committee of the same University. After approval by the Committee, the study will be registered on the Brazilian Registry of Clinical Trials (ReBEC) platform. After providing consent by signing the Informed Consent Form (Appendix B), people will be invited to come to LEPLO on five different days. On day 1, participants will undergo clinical, cognitive, and demographic assessments and familiarization with the treadmill (observation of

comfortable speed on the treadmill for each patient). On days 2, 3, 4 and 5, participants will sequentially perform the following experimental procedures: i) pre-intervention assessments (walking and cortical activity); ii) anodal tDCS session (M1, PFC, M1+PFC or sham) combined with treadmill training; and iii) post-intervention assessments (walking and cortical activity). The interval between intervention sessions will be one week to prevent the effects caused by one session from interfering with the others. Furthermore, the sequence of exposure to the sessions will be counterbalanced and randomized among participants in a 1:1:1:1 ratio using an online random number generator (www.randomization.com). To ensure the double-blind nature of the study, participants will not know which tDCS condition they will receive on each day of intervention and the member of the research team, responsible for manipulating the stimulation equipment, will not participate in the assessments (pre and post). All experimental procedures will be performed at the same time for each patient and all will be in the "ON" state of the specific PD medication, between 45 and 60 minutes after taking the medication.

3.3 Clinical assessment

An anamnesis will be applied to all participants to collect data on age, history of orthopedic and vestibular problems, medication, time since PD diagnosis and other relevant information. Anthropometric data will be collected, such as height and body mass. The history of falls over the last 12 months will be obtained from the LEPLO database. The daily dose of levodopa will be calculated following the recommendations of Tomlinson and colleagues (2010). Fear of falls and freezing will be assessed using the Falls Efficacy Scale – International (FES-I) (YARDLEY et al., 2005) and the Freezing of Gait Questionnaire (FOG-Q) (FOG-Q) (GILADI et al., 2009), respectively. The Movement Disorders Society – Unified Parkinson's Disease Rating Scale part III (MDS-UPDRS III) (GOETZ et al., 2008) will be applied to identify the degree of motor impairment in PD. Furthermore, the hemibody most affected by

PD will be determined using items from the MDS-UPDRS III (3.3b to 3.17d). The cerebral hemisphere most affected by PD will be inferred from the determination of the contralateral hemi body most affected. The adapted H&Y scale will be used to identify the evolutionary stage of the disease (GOETZ et al., 2004). The MMSE will be applied to track the global cognitive condition of people (BRUCKI et al., 2003). As a complement to the MMSE, parts A and B of the Trail Making Test will be applied to monitor people' mental flexibility, processing speed and executive function (TOMBAUGH, 2004), and the difference between parts B and A will be reported.

3.4 Gait evaluation

Initially, trained evaluators will place a headgear, which contains the fNIRS system, on the participant's head to record PFC activity. The positioning of the headgear will follow the international 10/20 system of electroencephalography (EEG), which consists of positioning the reference point of the headgear (Cz) at the midpoint between the nasion and inium and the pre-auricular points. The fNIRS optodes contained in the headgear will be positioned on the front of the head, corresponding to Brodmann areas 9, 10 and 46 of the left and right hemispheres, which represent the dorsolateral and anterior PFC (ORCIOLI-SILVA et al., 2020, 2021a). An accelerometer will be placed on the fifth lumbar vertebra to obtain the gait parameters. The height at which the accelerometer will be positioned on the lower back will be recorded. After these preparations, the participant will walk a 26.8m long circuit at preferred speed under the following conditions: i) walk at usual speed; ii) walking with a cognitive dual task; and iii) walking overcoming obstacles. Participants will perform 3 attempts in each of the experimental conditions. Each attempt will last a total of 60s. Initially, the participant will stand still for 30s and will be instructed to look straight ahead, remain silent and perform simple counts mentally (1 by 1 addition) to standardize the attentional demand during the baseline period. (HOLTZER

et al., 2015). After a verbal signal (“prepare, go”), the participant will perform the experimental condition for 30s. In the condition with overcoming obstacles, 4 obstacles made of foam (60cm long x 5cm wide x 15cm high) will be arranged along the circuit (ORCIOLI-SILVA et al., 2021b). In the cognitive dual task condition, an audio containing random numbers from 1 to 9 will play during the 30s of the walk and the participant must mentally count how many times a certain class of numbers (even or odd) was told (ORCIOLI-SILVA et al., 2020). The participant will be instructed on which class of numbers to count immediately before the start walking and will give his answer at the end of the attempt. The order of experimental conditions will be randomized for each individual. The equipment (fNIRS and accelerometer) will be removed to carry out the intervention protocol, but markings will be made to ensure the same positioning of the equipment in post-intervention evaluations.

3.5 Treadmill training with tDCS protocol

After pre-intervention assessments, the participant will be seated in a comfortable chair to measure blood pressure (auscultatory method), resting heart rate (HR) and to prepare for tDCS. Electrodes in sponges soaked in saline solution (35cm^2), connected to the Microestim Genius stimulator (NKL Produtos Eletrônicos Ltda. – EEP, Brusque/Santa Catarina, Brazil), will be placed in the target regions. Participants will perform four days of intervention with anodal tDCS: 1 day in M1, 1 day in PFC, 1 day in both areas simultaneously and 1 day will be sham/placebo. On the four days of intervention, the anode electrodes will be positioned in the M1 (position C3 or C4 of the 10-20 system) and in the PFC (position F3 or F4 of the 10-20 system) of the cerebral hemisphere most affected by PD. The cathode electrode (reference) will be positioned in the supraorbital region (position FP1 or FP2 of the 10-20 system) of the hemisphere contralateral to the anode electrodes. On days when stimulation is applied to only one area, the anode electrode will only be connected to M1 or PFC. The assembly of the

electrodes was designed in this way so that the people and the team member responsible for administering the intervention sessions do not know which tDCS condition is applied. In the three active tDCS sessions, the electrical current intensity will be 2mA for 20 min. Besides this period, there will be an initial 30s ramp-up (until reaching 2mA) and a final 30s ramp-down to 0 mA. In the sham tDCS session, there will be an initial 30s ramp-up, active stimulation (2mA) for 10s and a final 30s ramp-down to 0 mA. The equipment will be turned off for the remaining 19min and 50s.

Training on the ATL treadmill (Inbramed – Indústria Brasileira de Equipamentos Médicos Ltda., Porto Alegre/Rio Grande do Sul, Brazil) will last a total of 30 min and the intensity will be controlled by the maximum HR (HRmax). The training protocol will involve: i) 5 min warm-up with HR between 50-60% of HRmax; ii) main part of 20 min with intensity between 60-70% of HRmax, and iii) cool down for 5 min with HR below 60% of HRmax. The tDCS will be on (active or sham) in the main part of the training and off during the warm-up and cool down. The evaluator will increase and/or decrease the speed of the treadmill to maintain the HR within the established limits. HR will be monitored and recorded every minute using the Polar V800 heart rate monitor (Polar Electro Brasil Comércio, Distribuição, Importação e Exportação Ltda., São Paulo/SP, Brazil). HRmax will be estimated by the following equation: HRmax = 208 – 0.7*age (TANAKA; MONAHAN; SEALS, 2001). For participants taking beta-blocker medications, HRmax will be estimated by the equation: HRmax = 164 – 0.7*age (BRAWNER et al., 2004). Furthermore, the perceived exertion will be monitored every minute using the modified Borg scale (0-10 indexes) (BORG, 1982). For safety, a team member will be near the treadmill and the participant will be wearing a harness during the entire session. This equipment consists of a safety belt model CG 700/750, coupled to a retractable fall arrester model CG 500N (Carbografite Equipamentos Industriais Ltda., São Paulo/SP, Brazil), which will be attached to a pulley system fixed to the ceiling. Immediately

after the interventions, participants will be asked about sensations of discomfort and side effects caused by tDCS (BRUNONI et al., 2011), and blood pressure will be measured again.

3.6 Data analysis

3.6.1 Gait analysis

The gait parameters will be obtained through the acceleration of the center of mass. Data from the Trigno™ Avanti Sensor accelerometer will be transmitted via Wi-Fi to the base station of the Trigno™ Research+ system (Delsys, Inc., Natick Massachusetts, USA), and from there to a computer via cable. The accelerometer has a sampling frequency of 148.15 Hz and its data will be stored in the EMGworks® software (Delsys, Inc., Natick, Massachusetts, USA). The gait parameters will be obtained using algorithms in the MATLAB®R2015a environment (The MathWorks, Inc., Natick, Massachusetts, USA), previously validated by Del Din and collaborators (2016). Briefly, the acceleration data will be transformed using a vertical-horizontal coordinate system and will be filtered with a 4th order Butterworth filter (20 Hz) (MCCAMLEY et al., 2012; ZIJLSTRA; HOF, 2003). The initial and final contacts of the walking cycle will be estimated using the continuous wavelet transform (CWT) of vertical acceleration, which will first be integrated and then differentiated using a Gaussian CWT. The initial and final contact events will be detected through the minimum and maximum points of the CWT, respectively (DEL DIN; GODFREY; ROCHESTER, 2016). Both right and left foot (foot-to-ground) contacts will be identified. The initial and final contact times will be used to estimate the step duration and swing phase (DEL DIN; GODFREY; ROCHESTER, 2016). Step length will be determined from initial contact events by applying the inverted pendulum model described by Zijlstra and Hof (2003). Step speed and length, swing phase time, double support time and their respective variability (coefficient of variation = standard deviation/mean*100) will be analyzed. In the walking condition overcoming obstacles, contacts with obstacles will

be recorded and removed from the analysis. Cognitive task performance during dual-task walking will be quantified by absolute error (correct response – response spoken by the participant). All steps from the three trials per condition will be considered in the analysis.

3.6.2 Cortical activity analysis

PFC activity data will be acquired using a mobile fNIRS system (OctaMon, Artinis Medical System, Netherlands). An 8-channel arrangement with a sampling rate of 10 Hz. fNIRS is a non-invasive functional neuroimaging technique that records changes in the concentration of oxyhemoglobin (HbO2) and deoxyhemoglobin. Only HbO2 concentrations will be analyzed as this is a more sensitive indicator of cortical activity during walking (SUZUKI et al., 2004). The OctaMon model contains 10 optodes, 8 transmitters and 2 detectors (4 transmitters and 1 detector per hemisphere), which emit near-infrared lights of wavelengths of 760 to 850 nm. The distance between the optodes is 35 mm. The fNIRS signal amplifier will be positioned on the headgear itself (back of the neck) and this amplified signal will be sent in real time, via *Bluetooth*, to a computer. The OxySoft 3.0.52 software (Artinis Medical Systems, Elst, The Netherlands) will be used to collect, store and calculate HbO2 concentrations using the modified *Beer-Lambert* law.

The fNIRS signal analysis procedures (artifact corrections and filtering) will be performed using the opensource software NIRS-SPM (http://www.fil.ion.ucl.ac.uk/spm/software/) (YE et al., 2009) and will follow the recommendations of Vitório and collaborators (2017). Firstly, a wavelet-minimum description length filter (Wavelet MDL) (BRIGADOI et al., 2014; COOPER et al., 2012; JANG et al., 2009) will be used to decompose the signal into global trend, hemodynamic response and components of noise. Furthermore, high frequency noise will be reduced/removed using a low-pass filter based on the canonical function of the hemodynamic response (YE et al., 2009).

Then, customized algorithms written in the MATLAB® R2015a environment (The MathWorks, Inc., Natick, Massachusetts, USA) will be used to calculate the variables of interest. The HbO2 concentrations of the eight channels will be calculated, with four channels for each hemisphere. For data analysis, the task will be divided into two periods: baseline (last 10 seconds before the start of walking, when the participant is in a standing position) and walking (period between the fifth and twenty-fifth second after the start of walking). The average HbO2 concentration for each analyzed period and for each cerebral hemisphere (considering the average of all attempts performed per condition) will be calculated. The difference in HbO2 concentration between periods (walking – baseline) will be calculated to evaluate the relative change in cortical activity between walking and baseline (MAIDAN et al., 2016; MIRELMAN et al., 2014).

3.7 Statistical analysis

SPSS® 22.0 software (International Business Machines Corporation, Armonk, New York, USA) will be used for statistical treatment and the significance level will be maintained at < 0.05 for all analyses. The Shapiro-Wilk and Levene tests will be applied to verify the normality of data distribution and the homogeneity of variances, respectively. Data with normal distribution will be presented as mean ± standard deviation while data with non-normal distribution will be presented as median (quartiles 25 and 75). The deltas (Δ = post – pre) of the gait parameters and HbO2 concentration for each intervention session will be calculated. Then, two-way ANOVAs with repeated measures for the walking condition factors (usual X overcoming obstacle X cognitive dual task) and stimulation condition (M1 X PFC X M1+PFC X sham/placebo) will be applied. In case of interaction between factors, Bonferroni post hoc tests will be used to identify differences. The mean standardized response (MRP) will be used to verify the magnitude of the intervention in the gait parameters. MRP values will be

interpreted as trivial (<0.2), low (≥0.2), moderate (≥0.5) and high (≥0.8) (MIDDEL; VAN SONDEREN, 2002).

REFERENCES

ASCHERIO, Alberto; SCHWARZSCHILD, Michael A. The epidemiology of Parkinson's disease: risk factors and prevention. **The Lancet Neurology**, London, v. 15, n. 12, p. 1257–1272, 2016. DOI: 10.1016/S1474-4422(16)30230-7.

BARBOSA, Maira Tonidandel; CARAMELLI, Paulo; MAIA, Débora Palma; CUNNINGHAM, Mauro César Quintão; GUERRA, Henrique Leonardo; LIMA-COSTA, Maria Fernanda; CARDOSO, Francisco. Parkinsonism and Parkinson's disease in the elderly: A community-based survey in Brazil (the Bambuí study). **Movement Disorders**, New York, v. 21, n. 6, p. 800–808, 2006. DOI: 10.1002/mds.20806.

BENNINGER, David H.; LOMAREV, Mikhail; LOPEZ, Grisel; WASSERMANN, Eric M.; LI, Xiaobai; CONSIDINE, Elaine; HALLETT, Mark. Transcranial direct current stimulation for the treatment of Parkinson's disease. **Journal of Neurology, Neurosurgery & Psychiatry**, London, v. 81, n. 10, p. 1105–1111, 2010. DOI: 10.1136/jnnp.2009.202556.

BERETTA, Victor Spiandor; CONCEIÇÃO, Núbia Ribeiro; NÓBREGA-SOUSA, Priscila; ORCIOLI-SILVA, Diego; DANTAS, Luana Karla Braz Fonseca; GOBBI, Lilian Teresa Bucken; VITÓRIO, Rodrigo. Transcranial direct current stimulation combined with physical or cognitive training in people with Parkinson's disease: a systematic review. **Journal of NeuroEngineering and Rehabilitation**, London, v. 17, n. 1, p. 74, 2020. DOI: 10.1186/s12984-020-00701-6.

BORG, G. A. Psychophysical bases of perceived exertion. **Medicine and science in sports and exercise**, *[S. l.]*, v. 14, n. 5, p. 377–81, 1982.

BRAWNER, Clinton A.; EHRMAN, Jonathan K.; SCHAIRER, John R.; CAO, Jie J.; KETEYIAN, Steven J. Predicting maximum heart rate among people with coronary heart disease receiving beta-adrenergic blockade therapy. **American heart journal**, *[S. l.]*, v. 148, n. 5, p. 910–914, 2004. DOI: 10.1016/J.AHJ.2004.04.035.

BRIGADOI, Sabrina; CECCHERINI, Lisa; CUTINI, Simone; SCARPA, Fabio; SCATTURIN, Pietro; SELB, Juliette; GAGNON, Louis; BOAS, David A.; COOPER, Robert J. Motion artifacts in functional near-infrared spectroscopy: a comparison of motion correction techniques applied to real cognitive data. **NeuroImage**, *[S. l.]*, v. 85 Pt 1, n. 0 1, p. 181–191, 2014. DOI: 10.1016/J.NEUROIMAGE.2013.04.082.

BRUCKI, Sonia M. D.; NITRINI, Ricardo; CARAMELLI, Paulo; BERTOLUCCI, Paulo H. F.; OKAMOTO, Ivan H. Sugestões para o uso do mini-exame do estado mental no Brasil.

Arquivos de Neuro-Psiquiatria, *[S. l.]*, v. 61, n. 3B, p. 777–781, 2003. DOI: 10.1590/S0004-282X2003000500014.

BRUNONI, Andre Russowsky et al. Clinical research with transcranial direct current stimulation (tDCS): Challenges and future directions. **Brain Stimulation**, *[S. l.]*, v. 5, n. 3, p. 175–195, 2012. DOI: 10.1016/j.brs.2011.03.002.

BRUNONI, Andre Russowsky; AMADERA, Joao; BERBEL, Bruna; VOLZ, Magdalena Sarah; RIZZERIO, Brenno Gomes; FREGNI, Felipe. A systematic review on reporting and assessment of adverse effects associated with transcranial direct current stimulation. **International Journal of Neuropsychopharmacology**, *[S. l.]*, v. 14, n. 8, p. 1133–1145, 2011. DOI: 10.1017/S1461145710001690.

BUENO, Maria Eduarda Brandão; DO NASCIMENTO NETO, Luiz Inácio; TERRA, Marcelle Brandão; BARBOZA, Natália Mariano; OKANO, Alexandre Hideki; SMAILI, Suhaila Mahmoud. Effectiveness of acute transcranial direct current stimulation on non-motor and motor symptoms in Parkinson’s disease. **Neuroscience Letters**, *[S. l.]*, v. 696, p. 46–51, 2019. DOI: 10.1016/j.neulet.2018.12.017.

CONCEIÇÃO, Núbia Ribeiro; GOBBI, Lilian Teresa Bucken; NÓBREGA-SOUSA, Priscila; ORCIOLI-SILVA, Diego; BERETTA, Victor Spiandor; LIRANI-SILVA, Ellen; OKANO, Alexandre Hideki; VITÓRIO, Rodrigo. Aerobic Exercise Combined With Transcranial Direct Current Stimulation Over the Prefrontal Cortex in Parkinson Disease: Effects on Cortical Activity, Gait, and Cognition. **Neurorehabilitation and Neural Repair**, *[S. l.]*, v. 35, n. 8, p. 717–728, 2021. DOI: 10.1177/15459683211019344.

COOPER, Robert J.; SELB, Juliette; GAGNON, Louis; PHILLIP, Dorte; SCHYTZ, Henrik W.; IVERSEN, Helle K.; ASHINA, Messoud; BOAS, David A. A systematic comparison of motion artifact correction techniques for functional near-infrared spectroscopy. **Frontiers in neuroscience**, *[S. l.]*, v. 6, n. OCT, 2012. DOI: 10.3389/FNINS.2012.00147.

COSTA-RIBEIRO, Adriana et al. Transcranial direct current stimulation associated with gait training in Parkinson’s disease: A pilot randomized clinical trial. **Developmental Neurorehabilitation**, *[S. l.]*, v. 20, n. 3, p. 121–128, 2017. DOI: 10.3109/17518423.2015.1131755.

CURTZE, Carolin; NUTT, John G.; CARLSON-KUHTA, Patricia; MANCINI, Martina; HORAK, Fay B. Levodopa Is a Double-Edged Sword for Balance and Gait in People With Parkinson’s Disease. **Movement Disorders**, New York, v. 30, n. 10, p. 1361–1370, 2015. DOI: 10.1002/mds.26269.

DA SILVA, Débora Cristina Lima; LEMOS, Thiago; DE SÁ FERREIRA, Arthur; HORSCZARUK, Carlos Henrique Ramos; PEDRON, Carla Andressa; DE CARVALHO RODRIGUES, Erika; DE OLIVEIRA, Laura Alice Santos. Effects of Acute Transcranial Direct Current Stimulation on Gait Kinematics of Individuals With Parkinson Disease. **Topics in Geriatric Rehabilitation**, *[S. l.]*, v. 34, n. 4, p. 262–268, 2018. DOI: 10.1097/TGR.0000000000000203.

DAGAN, Moria; HERMAN, Talia; HARRISON, Rachel; ZHOU, Junhong; GILADI, Nir; RUFFINI, Giulio; MANOR, Brad; HAUSDORFF, Jeffrey M. Multitarget transcranial direct current stimulation for freezing of gait in Parkinson's disease. **Movement Disorders**, *[S. l.]*, v. 33, n. 4, p. 642–646, 2018. DOI: 10.1002/mds.27300.

DEL DIN, Silvia; GODFREY, Alan; ROCHESTER, Lynn. Validation of an Accelerometer to Quantify a Comprehensive Battery of Gait Characteristics in Healthy Older Adults and Parkinson's Disease: Toward Clinical and at Home Use. **IEEE Journal of Biomedical and Health Informatics**, *[S. l.]*, v. 20, n. 3, p. 838–847, 2016. DOI: 10.1109/JBHI.2015.2419317.

DORUK, Deniz; GRAY, Zachary; BRAVO, Gabriela L.; PASCUAL-LEONE, Alvaro; FREGNI, Felipe. Effects of tDCS on executive function in Parkinson's disease. **Neuroscience Letters**, *[S. l.]*, v. 582, p. 27–31, 2014. DOI: 10.1016/j.neulet.2014.08.043.

FREGNI, Felipe et al. Noninvasive cortical stimulation with transcranial direct current stimulation in Parkinson's disease. **Movement Disorders**, New York, v. 21, n. 10, p. 1693–1702, 2006. DOI: 10.1002/mds.21012.

GALNA, Brook; MURPHY, Anna T.; MORRIS, Meg E. Obstacle crossing in people with Parkinson's disease: Foot clearance and spatiotemporal deficits. **Human Movement Science**, *[S. l.]*, v. 29, n. 5, p. 843–852, 2010. DOI: 10.1016/j.humov.2009.09.006.

GALVAN, Adriana; WICHMANN, Thomas. Pathophysiology of parkinsonism. **Clinical neurophysiology : official journal of the International Federation of Clinical Neurophysiology**, *[S. l.]*, v. 119, n. 7, p. 1459–1474, 2008. DOI: 10.1016/J.CLINPH.2008.03.017.

GBD 2016 PARKINSON'S DISEASE COLLABORATORS. Global, regional, and national burden of Parkinson's disease, 1990–2016: a systematic analysis for the Global Burden of Disease Study 2016. **The Lancet Neurology**, London, v. 17, n. 11, p. 939–953, 2018. DOI: 10.1016/S1474-4422(18)30295-3.

GILADI, Nir et al. Validation of the freezing of gait questionnaire in people with Parkinson's disease. **Movement Disorders**, New York, v. 24, n. 5, p. 655–661, 2009. DOI: 10.1002/mds.21745.

GOETZ, Christopher G. et al. Movement Disorder Society Task Force report on the Hoehn and Yahr staging scale: Status and recommendations The Movement Disorder Society Task Force on rating scales for Parkinson's disease. **Movement Disorders**, New York, v. 19, n. 9, p. 1020–1028, 2004. DOI: 10.1002/mds.20213.

GOETZ, Christopher G. et al. Movement Disorder Society-sponsored revision of the Unified Parkinson's Disease Rating Scale (MDS-UPDRS): Scale presentation and clinimetric testing results. **Movement Disorders**, New York, v. 23, n. 15, p. 2129–2170, 2008. DOI: 10.1002/mds.22340.

HEROLD, Fabian; WIEGEL, Patrick; SCHOLKMANN, Felix; THIERS, Angelina; HAMACHER, Dennis; SCHEGA, Lutz. Functional near-infrared spectroscopy in movement science: a systematic review on cortical activity in postural and walking tasks. **Neurophotonics**, *[S. l.]*, v. 4, n. 4, p. 041403, 2017. DOI: 10.1117/1.NPh.4.4.041403.

HOLTZER, Roee; MAHONEY, Jeannette R.; IZZETOGLU, Meltem; WANG, Cuiling; ENGLAND, Sarah; VERGHESE, Joe. Online fronto-cortical control of simple and attention-demanding locomotion in humans. **NeuroImage**, *[S. l.]*, v. 112, p. 152–159, 2015. DOI: 10.1016/j.neuroimage.2015.03.002.

HUGHES, Andrew J.; DANIEL, Susan E.; KILFORD, Linda; LEES, Andrew J. Accuracy of clinical diagnosis of idiopathic Parkinson’s disease: a clinico-pathological study of 100 cases. **Journal of Neurology, Neurosurgery & Psychiatry**, London, v. 55, n. 3, p. 181–184, 1992. DOI: 10.1136/jnnp.55.3.181.

JANG, Kwang-Eun; TAK, Sungho; JUNG, Jinwook; JANG, Jaeduck; JEONG, Yong; YE, Yong Chul. Wavelet minimum description length detrending for near-infrared spectroscopy. **https://doi.org/10.1117/1.3127204**, *[S. l.]*, v. 14, n. 3, p. 034004, 2009. DOI: 10.1117/1.3127204.

JANKOVIC, Joseph. Parkinson’s disease therapy: tailoring choices for early and late disease, young and old people. **Clinical Neuropharmacology**, *[S. l.]*, v. 23, n. 5, p. 252–61, 2000. DOI: 10.1097/00002826-200009000-00003.

KASKI, D.; ALLUM, J. H.; BRONSTEIN, A. M.; DOMINGUEZ, R. O. Applying anodal tDCS during tango dancing in a patient with Parkinson’s disease. **Neuroscience Letters**, *[S. l.]*, v. 568, p. 39–43, 2014. a. DOI: 10.1016/j.neulet.2014.03.043.

KASKI, D.; DOMINGUEZ, RO; ALLUM, JH; ISLAM, AF; BRONSTEIN, AM. Combining physical training with transcranial direct current stimulation to improve gait in Parkinson’s disease: a pilot randomized controlled study. **Clinical Rehabilitation**, *[S. l.]*, v. 28, n. 11, p. 1115–1124, 2014. b. DOI: 10.1177/0269215514534277.

KELLY, Valerie E.; EUSTERBROCK, Alexis J.; SHUMWAY-COOK, Anne. A Review of Dual-Task Walking Deficits in People with Parkinson’s Disease: Motor and Cognitive Contributions, Mechanisms, and Clinical Implications. **Parkinson’s Disease**, *[S. l.]*, v. 2012, p. 1–14, 2012. DOI: 10.1155/2012/918719.

LEE, Hyo Keun; AHN, Se Ji; SHIN, Yang Mi; KANG, Nyeonju; CAURAUGH, James H. Does transcranial direct current stimulation improve functional locomotion in people with Parkinson’s disease? A systematic review and meta-analysis. **Journal of NeuroEngineering and Rehabilitation**, *[S. l.]*, v. 16, n. 1, p. 84, 2019. DOI: 10.1186/s12984-019-0562-4.

MAIDAN, Inbal et al. The role of the frontal lobe in complex walking among people With Parkinson’s Disease and healthy older adults: An fNIRS study. **Neurorehabilitation and Neural Repair**, New York, v. 30, n. 10, p. 963–971, 2016. DOI: 10.1177/1545968316650426.

MCCAMLEY, John; DONATI, Marco; GRIMPAMPI, Eleni; MAZZÀ, Claudia. An enhanced estimate of initial contact and final contact instants of time using lower trunk inertial sensor data. **Gait & Posture**, *[S. l.]*, v. 36, n. 2, p. 316–318, 2012. DOI: 10.1016/j.gaitpost.2012.02.019.

MEHRHOLZ, Jan; KUGLER, Joachim; STORCH, Alexander; POHL, Marcus; HIRSCH, Kathleen; ELSNER, Bernhard. Treadmill training for people with Parkinson's disease. **Cochrane Database of Systematic Reviews**, *[S. l.]*, v. 2015, n. 9, 2015. DOI: 10.1002/14651858.CD007830.pub4.

MIDDEL, Berrie; VAN SONDEREN, Eric. Statistical significant change versus relevant or important change in (quasi) experimental design: some conceptual and methodological problems in estimating magnitude of intervention-related change in health services research. **International Journal of Integrated Care**, *[S. l.]*, v. 2, n. 4, 2002. DOI: 10.5334/ijic.65.

MIRELMAN, Anat et al. Gait impairments in Parkinson's disease. **The Lancet Neurology**, London, v. 18, n. 7, p. 697–708, 2019. DOI: 10.1016/S1474-4422(19)30044-4.

MIRELMAN, Anat; MAIDAN, Inbal; BERNAD-ELAZARI, Hagar; NIEUWHOF, Freek; REELICK, Miriam; GILADI, Nir; HAUSDORFF, M. Jeffrey. Increased frontal brain activation during walking while dual tasking: An fNIRS study in healthy young adults. **Journal of NeuroEngineering and Rehabilitation**, *[S. l.]*, v. 11, n. 1, p. 1–7, 2014. DOI: 10.1186/1743-0003-11-85.

MISHRA, Ram kinker; THRASHER, Adam T. Transcranial direct current stimulation of dorsolateral prefrontal cortex improves dual-task gait performance in people with Parkinson's disease: A double blind, sham-controlled study. **Gait & Posture**, *[S. l.]*, v. 84, p. 11–16, 2021. DOI: 10.1016/j.gaitpost.2020.11.012.

NIEUWHOF, Freek; REELICK, Miriam F.; MAIDAN, Inbal; MIRELMAN, Anat; HAUSDORFF, Jeffrey M.; OLDE RIKKERT, Marcel G. M.; BLOEM, Bastiaan R.; MUTHALIB, Makii; CLAASSEN, Jurgen A. H. R. Measuring prefrontal cortical activity during dual task walking in people with Parkinson's disease: feasibility of using a new portable fNIRS device. **Pilot and Feasibility Studies**, *[S. l.]*, v. 2, n. 1, p. 59, 2016. DOI: 10.1186/s40814-016-0099-2.

NITSCHE, M. A.; LIEBETANZ, D.; TERGAU, F.; PAULUS, W. Modulation of cortical excitability by transcranial direct current stimulation. **Der Nervenarzt**, *[S. l.]*, v. 73, n. 4, p. 332–335, 2002. DOI: 10.1007/s00115-002-1272-9.

NITSCHE, Michael A. et al. Transcranial direct current stimulation: State of the art 2008. **Brain Stimulation**, *[S. l.]*, v. 1, n. 3, p. 206–223, 2008. DOI: 10.1016/j.brs.2008.06.004.

NONNEKES, Jorik; TIMMER, Monique H. M.; DE VRIES, Nienke M.; RASCOL, Olivier; HELMICH, Rick C.; BLOEM, Bastiaan R. Unmasking levodopa resistance in Parkinson's disease. **Movement Disorders**, *[S. l.]*, v. 31, n. 11, p. 1602–1609, 2016. DOI: 10.1002/mds.26712.

ORCIOLI-SILVA, Diego; VITÓRIO, Rodrigo; BERETTA, Victor Spiandor; DA CONCEIÇÃO, Núbia Ribeiro; NÓBREGA-SOUSA, Priscila; OLIVEIRA, Anderson Souza; GOBBI, Lilian Teresa Bucken. Is Cortical Activation During Walking Different Between Parkinson's Disease Motor Subtypes? **The Journals of Gerontology: Series A**, *[S. l.]*, v. 76, n. 4, p. 561–567, 2021. a. DOI: 10.1093/gerona/glaa174.

ORCIOLI-SILVA, Diego; VITÓRIO, Rodrigo; NÓBREGA-SOUSA, Priscila; BERETTA, Victor Spiandor; CONCEIÇÃO, Núbia Ribeiro Da; OLIVEIRA, Anderson Souza; PEREIRA, Marcelo Pinto; GOBBI, Lilian Teresa Bucken. Cortical Activity Underlying Gait Improvements Achieved With Dopaminergic Medication During Usual Walking and Obstacle Avoidance in Parkinson Disease. **Neurorehabilitation and Neural Repair**, *[S. l.]*, v. 35, n. 5, p. 406–418, 2021. b. DOI: 10.1177/15459683211000736.

ORCIOLI-SILVA, Diego; VITÓRIO, Rodrigo; NÓBREGA-SOUSA, Priscila; DA CONCEIÇÃO, Núbia Ribeiro; BERETTA, Victor Spiandor; LIRANI-SILVA, Ellen; GOBBI, Lilian Teresa Bucken. Levodopa Facilitates Prefrontal Cortex Activation During Dual Task Walking in Parkinson Disease. **Neurorehabilitation and Neural Repair**, *[S. l.]*, v. 34, n. 7, p. 589–599, 2020. DOI: 10.1177/1545968320924430.

PETERSEN, T. H.; WILLERSLEV-OLSEN, M.; CONWAY, B. A.; NIELSEN, J. B. The motor cortex drives the muscles during walking in human subjects. **The Journal of Physiology**, *[S. l.]*, v. 590, n. 10, p. 2443–2452, 2012. DOI: 10.1113/jphysiol.2012.227397. Disponível em: http://doi.wiley.com/10.1113/jphysiol.2012.227397.

PETERSON, D. S.; HORAK, F. B. Neural Control of Walking in People with Parkinsonism. **Physiology**, *[S. l.]*, v. 31, n. 2, p. 95–107, 2016. DOI: 10.1152/physiol.00034.2015.

POL, Fateme; SALEHINEJAD, Mohammad Ali; BAHARLOUEI, Hamzeh; NITSCHE, Michael A. The effects of transcranial direct current stimulation on gait in people with Parkinson's disease: a systematic review. **Translational Neurodegeneration**, *[S. l.]*, v. 10, n. 1, p. 22, 2021. DOI: 10.1186/s40035-021-00245-2.

ROCHESTER, Lynn; BAKER, Katherine; NIEUWBOER, Alice; BURN, David. Targeting dopa-sensitive and dopa-resistant gait dysfunction in Parkinson's disease: Selective responses to internal and external cues. **Movement Disorders**, *[S. l.]*, v. 26, n. 3, p. 430–435, 2011. DOI: 10.1002/mds.23450.

STUART, Samuel; BELLUSCIO, Valeria; QUINN, Joseph F.; MANCINI, Martina. Pre-frontal Cortical Activity During Walking and Turning Is Reliable and Differentiates Across Young, Older Adults and People With Parkinson's Disease. **Frontiers in Neurology**, *[S. l.]*, v. 10, n. MAY, 2019. DOI: 10.3389/fneur.2019.00536.

SUZUKI, Mitsuo; MIYAI, Ichiro; ONO, Takeshi; ODA, Ichiro; KONISHI, Ikuo; KOCHIYAMA, Takanori; KUBOTA, Kisou. Prefrontal and premotor cortices are involved in adapting walking and running speed on the treadmill: an optical imaging study. **NeuroImage**, *[S. l.]*, v. 23, n. 3, p. 1020–6, 2004. DOI: 10.1016/j.neuroimage.2004.07.002.

TAKAKUSAKI, Kaoru; TOMITA, Nozomi; YANO, Masafumi. Substrates for normal gait and pathophysiology of gait disturbances with respect to the basal ganglia dysfunction. **Journal of Neurology**, *[S. l.]*, v. 255, n. S4, p. 19–29, 2008. DOI: 10.1007/s00415-008-4004-7

TANAKA, Hirofumi; MONAHAN, Kevin D.; SEALS, Douglas R. Age-predicted maximal heart rate revisited. **Journal of the American College of Cardiology**, *[S. l.]*, v. 37, n. 1, p. 153–156, 2001. DOI: 10.1016/S0735-1097(00)01054-8.

TARAZI, F. I.; SAHLI, Z. T.; WOLNY, M.; MOUSA, S. A. Emerging therapies for Parkinson's disease: from bench to bedside. **Pharmacology & therapeutics**, *[S. l.]*, v. 144, n. 2, p. 123–33, 2014. DOI: 10.1016/j.pharmthera.2014.05.010.

TOMBAUGH, T. Trail Making Test A and B: Normative data stratified by age and education. **Archives of Clinical Neuropsychology**, *[S. l.]*, v. 19, n. 2, p. 203–214, 2004. DOI: 10.1016/S0887-6177(03)00039-8.

TOMLINSON, Claire L.; STOWE, Rebecca; PATEL, Smitaa; RICK, Caroline; GRAY, Richard; CLARKE, Carl E. Systematic review of levodopa dose equivalency reporting in Parkinson's disease. **Movement Disorders**, New York, v. 25, n. 15, p. 2649–2653, 2010. DOI: 10.1002/mds.23429.

VITORIO, R.; STUART, S.; ROCHESTER, L.; ALCOCK, L.; PANTALL, A. fNIRS response during walking — Artefact or cortical activity? A systematic review. **Neuroscience & Biobehavioral Reviews**, *[S. l.]*, v. 83, p. 160–172, 2017. DOI: 10.1016/j.neubiorev.2017.10.002.

VITÓRIO, Rodrigo; LIRANI-SILVA, Ellen; BAPTISTA, André Macari; BARBIERI, Fabio Augusto; DOS SANTOS, Paulo Cezar Rocha; TEIXEIRA-ARROYO, Claudia; GOBBI, Lilian Teresa Bucken. Disease severity affects obstacle crossing in people with Parkinson's disease. **Gait & Posture**, *[S. l.]*, v. 40, n. 1, p. 266–269, 2014. DOI: 10.1016/j.gaitpost.2014.03.003.

VITÓRIO, Rodrigo; PIERUCCINI-FARIA, Frederico; STELLA, Florindo; GOBBI, Sebastião; GOBBI, Lilian Teresa Bucken. Effects of obstacle height on obstacle crossing in mild Parkinson's disease. **Gait & Posture**, *[S. l.]*, v. 31, n. 1, p. 143–146, 2010. DOI: 10.1016/j.gaitpost.2009.09.011.

WICHMANN, Thomas; DELONG, Mahlon R. Anatomy and physiology of the basal ganglia: relevance to Parkinson's disease and related disorders. *In*: **Handbook of clinical neurology**. [s.l.] : Handb Clin Neurol, 2007. v. 83p. 1–18. DOI: 10.1016/S0072-9752(07)83001-6.

YARDLEY, Lucy; BEYER, Nina; HAUER, Klaus; KEMPEN, Gertrudis; PIOT-ZIEGLER, Chantal; TODD, Chris. Development and initial validation of the Falls Efficacy Scale-International (FES-I). **Age and Ageing**, London, v. 34, n. 6, p. 614–619, 2005. DOI: 10.1093/ageing/afi196.

YE, Jong Chul; TAK, Sungho; JANG, Kwang Eun; JUNG, Jinwook; JANG, Jaeduck. NIRS-SPM: statistical parametric mapping for near-infrared spectroscopy. **NeuroImage**, *[S. l.]*, v. 44, n. 2, p. 428–447, 2009. DOI: 10.1016/J.NEUROIMAGE.2008.08.036.

YOTNUENGNIT, Pattarapol; BHIDAYASIRI, Roongroj; DONKHAN, Rattana; CHALUAYSRIMUANG, Juthamas; PIRAVEJ, Krisna. Effects of Transcranial Direct Current Stimulation Plus Physical Therapy on Gait in People With Parkinson Disease. **American Journal of Physical Medicine & Rehabilitation**, *[S. l.]*, v. 97, n. 1, p. 7–15, 2018. DOI: 10.1097/PHM.0000000000000783.

ZIJLSTRA, Wiebren; HOF, At L. Assessment of spatio-temporal gait parameters from trunk accelerations during human walking. **Gait & Posture**, *[S. l.]*, v. 18, n. 2, p. 1–10, 2003. DOI: 10.1016/S0966-6362(02)00190-X.
