# Supplementary material for: Aerobic exercise on the treadmill combined with transcranial direct current stimulation on the gait of people with Parkinson’s disease: A protocol for a randomized clinical trial
Source: PLoS One. 2024 Apr 25;19(4):e0300243. doi: 10.1371/journal.pone.0300243 (PMC11045059; doi:10.1371/journal.pone.0300243)
Supplement: S2 File — (PDF) [file pone.0300243.s003.pdf]

EFEITOS DA ESTIMULAÇÃO TRANSCRANIANA POR CORRENTE CONTÍNUA COMBINADA COM TREINO EM ESTEIRA NO ANDAR DE PACIENTES COM DOENÇA DE PARKINSON: UM ENSAIO CLÍNICO RANDOMIZADO

Pesquisador Responsável: Gabriel Antonio Gazziero Moraca

Equipe de Pesquisa: Profa. Dra. Lilian Teresa Bucken Gobbi; Prof. Dr. Diego Orcioli-Silva; Prof. Me. Victor Spiandor Beretta e a graduanda Beatriz Regina Legutke.

Instituição sede: Universidade Estadual Paulista (Unesp), Instituto de Biociências, Rio Claro

RESUMO

Comprometimentos no andar prejudicam a qualidade de vida de pacientes com doença de Parkinson (DP). A estimulação transcraniana por corrente contínua (ETCC) vem mostrando resultados inconclusivos no andar, mas evidências sugerem que a combinação de ETCC com exercício físico pode ser benéfica para o andar dos pacientes com DP. O objetivo geral desta pesquisa é verificar os efeitos agudos da ETCC anódica aplicada no córtex motor primário (M1) e no córtex pré-frontal (PFC) combinada com treino em esteira no andar de pacientes com DP. Esta pesquisa será um ensaio clínico randomizado crossover, duplo-cego e sham-controlado. Dez pacientes com DP serão recrutados para participar deste estudo por cinco dias no total. No dia 1, os pacientes realizarão avaliações clínicas, cognitivas e familiarização na esteira (observação da velocidade confortável na esteira). Nos dias 2, 3, 4 e 5, os pacientes participarão de avaliações pré e pós-intervenção (andar e atividade cortical) e intervenção combinada de ETCC anódica com treino em esteira. O intervalo entre os dias de intervenção será de uma semana e a sequência de exposição às sessões será randomizada entre os pacientes. A ETCC ativa será aplicada no M1 e no PFC (separada e simultaneamente) e haverá uma sessão de ETCC sham/placebo. O treino na esteira será composto por: i) aquecimento de 5 min; ii) parte principal de 20 min e; iii) volta à calma de 5 min. Por segurança, os pacientes estarão com um equipamento arnês durante todo o tempo do treino. A intensidade do treino será controlada pela frequência cardíaca máxima de cada paciente. Durante o treino na esteira, os pacientes receberão a ETCC ativa por 20 min ou a ETCC sham por 10s (desligada nos minutos subsequentes). Antes e após as sessões de intervenção, os pacientes andarão em um circuito de 26,8m de comprimento em velocidade preferida nas seguintes condições: i) usual; ii) com ultrapassagem de obstáculos e; iii) com tarefa dupla cognitiva. Haverá 3 tentativas em cada condição e um acelerômetro posicionado na quinta vértebra lombar será utilizado para obtenção das variáveis do andar. Velocidade e comprimento do passo, duração da fase de balanço e do

duplo suporte e suas respectivas variabilidades serão analisadas. Atividade do córtex pré-frontal durante o andar será registrada por meio da espectroscopia funcional de luz próxima ao infravermelho e as concentrações de oxi-hemoglobina (HbO2) de ambos os hemisférios serão analisadas. Os deltas das variáveis do andar e da concentração de HbO2 de cada sessão de intervenção serão calculados. ANOVAs two-way com medidas repetidas para os fatores condição do andar (usual X com ultrapassagem de obstáculo X com tarefa dupla cognitiva) e condição de estimulação (M1 X PFC X M1+PFC X sham) serão empregadas. Testes post hoc de Bonferroni serão aplicados para identificar as diferenças. O nível de significância será mantido em < 0,05 e a média de resposta padronizada será utilizada para verificar a magnitude da intervenção nas variáveis do andar.

Palavras-chave: Estimulação cerebral. Exercício físico agudo. Locomoção. Andar adaptativo. Dupla tarefa. Doenças neurodegenerativas.

1 CONTEXTUALIZAÇÃO E JUSTIFICATIVA DA PROPOSTA

A estimulação transcraniana por corrente contínua (ETCC) é uma técnica que tem se mostrado eficiente em melhorar os sintomas motores da doença de Parkinson (DP), tais como: bradicinesia (BENNINGER et al., 2010) e rigidez (FREGNI et al., 2006). Entretanto, os estudos com relação aos efeitos da ETCC no andar de pacientes com DP ainda são inconclusivos. Por exemplo, um estudo recente mostrou diminuição da cadência, mas não encontrou aumento da velocidade e do comprimento da passada após ETCC anódica no córtex motor primário (M1) e na área motora suplementar (DA SILVA et al., 2018). Em contrapartida, Mishra e colaboradores (2021) encontraram aumento da velocidade do andar após ETCC anódica aplicada no córtex pré-frontal (PFC). Uma possível explicação para estes resultados controversos é a aplicação de diferentes protocolos de ETCC (intensidade, duração, região alvo etc.). Além disso, outros trabalhos combinaram a ETCC com diferentes intervenções motoras para melhorar o andar de pacientes com DP (KASKI et al., 2014a, 2014b; YOTNUENGNIT et al., 2018). Entretanto, os resultados continuam inconsistentes. Vale ressaltar que aplicar a ETCC com intervenção (motora e/ou cognitiva) concomitante, parece ser uma estratégia promissora para melhorar os déficits motores gerados pela DP (BERETTA et al., 2020). Portanto, avançar o conhecimento sobre os possíveis benefícios da ETCC combinada com uma intervenção motora no andar de pacientes com DP é necessário.

O nosso grupo de pesquisa, o Laboratório de Estudos da Postura e da Locomoção (LEPLO), tem se dedicado a investigar os efeitos da ETCC com ou sem intervenção motora concomitante nos déficits motores dos pacientes com DP. Neste sentido, um estudo recente mostrou que a ETCC anódica aplicada no PFC combinada com exercício em ciclo ergômetro melhora o tempo de reação e atividade cortical durante o andar, mas não as variáveis do andar (CONCEIÇÃO et al., 2021). Este estudo teve limitações, como estimular apenas uma área (PFC) e utilizar o ciclo ergômetro. Uma meta-análise recente sugere que estimular mais de uma

área cortical (ao mesmo tempo ou não) é mais benéfico para o controle do andar comparado à estimulação em apenas uma área (LEE et al., 2019). Ainda, treinamento em esteira pode acarretar benefícios mais significativos por encorajar o gesto motor similar ao andar. A presente proposta busca avançar o conhecimento dos efeitos desta técnica, aplicando-a em mais de uma região cortical (PFC e M1) concomitantemente com treino em esteira, no andar em diferentes dificuldades (por exemplo, andar usual, andar com tarefa dupla e andar com ultrapassagem de obstáculos). Vale destacar que o treino em esteira gera benefícios no andar (MEHRHOLZ et al., 2015) e que a combinação desta forma de intervenção com a ETCC foi pouca explorada em pacientes com DP até o momento.

A presente proposta possui impactos científicos, tecnológicos, na saúde e educacionais. Em relação ao impacto científico, a presente proposta visa progredir o conhecimento sobre os efeitos da ETCC combinada com exercício físico aeróbio em esteira no andar de pacientes com DP. Os resultados da presente proposta serão divulgados das seguintes maneiras: comunicações orais e/ou pôsteres em eventos científicos nas áreas de Comportamento Motor e Neurociências e artigos científicos em periódicos com fator de impacto maior do que 3 (por exemplo, *Neurorehabilitation & Neural Repair* e/ou *Parkinsonism & Related Disorders*). A competência técnica que será desenvolvida pelos membros da pesquisa, no uso de equipamentos portáteis que avaliam o controle da andar em "tempo real" de maneira biomecânica (acelerômetro) e cortical (espectroscopia funcional de luz próxima ao infravermelho – fNIRS), somados à aplicação de estimulação cerebral não-invasiva (ETCC), representam o impacto tecnológico da proposta. Em relação ao impacto na saúde e relacionado ao Objetivo de Desenvolvimento Sustentável nº 3 da Organização das Nações Unidas, os achados decorrentes da presente proposta irão contribuir para otimização de programas de intervenção focados na melhora do andar em pacientes com DP. Os impactos educacionais referem-se ao desenvolvimento do candidato como pesquisador e dos demais membros do LEPLO, no sentido de desenvolver

competências de coleta, análise de dados e discussão sobre atividade cortical, estimulação cerebral e exercício físico e à transferência do conhecimento produzido para as atividades de ensino de graduação e pós-graduação. Portanto, a presente proposta, além de contribuir para o entendimento dos efeitos da ETCC combinada com exercício no andar de pacientes com DP, também representa ganhos na formação do candidato, nas pesquisas desenvolvidas no LEPLO e na saúde da população investigada.

2 INTRODUÇÃO

A DP é a segunda doença neurodegenerativa mais prevalente no mundo (ASCHERIO; SCHWARZSCHILD, 2016), sendo que o número de casos mais do que dobrou nos últimos 30 anos (2,5 milhões de pessoas em 1990 contra 6,1 milhões de indivíduos em 2016) (GBD 2016 PARKINSON'S DISEASE COLLABORATORS, 2018). Especificamente no Brasil, a DP atinge cerca de 3,3% dos indivíduos acima dos 64 anos de idade (BARBOSA et al., 2006). A característica fisiopatológica da DP é a degeneração dos neurônios dopaminérgicos da substância negra parte compacta, responsáveis pela produção de dopamina (GALVAN; WICHMANN, 2008; WICHMANN; DELONG, 2007). A perda da dopamina provoca desequilíbrio nos sinais inibitórios e excitatórios que são enviados pelos núcleos da base ao córtex motor (PETERSON; HORAK, 2016; TAKAKUSAKI; TOMITA; YANO, 2008). Como consequência deste desequilíbrio, os pacientes com DP apresentam os seguintes comprometimentos motores: rigidez muscular, tremor de repouso, bradicinesia (lentidão dos movimentos), hipometria (redução da amplitude dos movimentos), instabilidade postural e comprometimentos no andar (GALVAN; WICHMANN, 2008).

O andar de pacientes com DP é caracterizado pela diminuição da velocidade do andar, do comprimento do passo e da fase de balanço, e aumento do tempo em duplo suporte e da variabilidade do andar (MIRELMAN et al., 2019). Além disso, os déficits no andar dos

pacientes são exacerbados em situações mais complexas, como durante o andar com ultrapassagem de obstáculos (GALNA; MURPHY; MORRIS, 2010; VITÓRIO et al., 2010, 2014) ou andar realizando uma tarefa cognitiva concomitantemente (tarefa dupla) (KELLY; EUSTERBROCK; SHUMWAY-COOK, 2012; ORCIOLI-SILVA et al., 2020). Recentemente, estudos têm identificado alterações em níveis corticais durante o andar em pacientes com DP. Por exemplo, estudos usando a fNIRS observaram que pacientes com DP apresentam maior atividade do PFC durante o andar usual comparado aos idosos sadios (MAIDAN et al., 2016; STUART et al., 2019). Ainda, a atividade do PFC é aumentada durante o andar com ultrapassagem de obstáculos (MAIDAN et al., 2016; ORCIOLI-SILVA et al., 2021b) e com tarefa dupla (NIEUWHOF et al., 2016; ORCIOLI-SILVA et al., 2020) comparado ao andar usual em pacientes com DP. Este aumento na atividade cortical durante o andar indica que os pacientes usam mecanismos compensatórios para lidar com os comprometimentos do andar (HEROLD et al., 2017). Sendo assim, investigar formas de intervenção que melhorem o andar em diversas condições presentes no dia a dia dos pacientes é clinicamente importante.

O tratamento mais utilizado para amenizar os sintomas da DP é o farmacológico, por meio do uso de levodopa – precursora da dopamina (NONNEKES et al., 2016; TARAZI et al., 2014). O tratamento farmacológico melhora alguns sintomas motores da DP, como tremor de repouso e a rigidez (NONNEKES et al., 2016), e melhora a velocidade do andar e o comprimento da passada (CURTZE et al., 2015; ORCIOLI-SILVA et al., 2020). Ainda, o tratamento farmacológico facilita o recrutamento do PFC durante o andar com ultrapassagem de obstáculos (ORCIOLI-SILVA et al., 2021b) e com tarefa dupla (ORCIOLI-SILVA et al., 2020). Entretanto, o uso prolongado do medicamento acarreta flutuações motoras, discinesias (movimentos involuntários) (JANKOVIC, 2000; NONNEKES et al., 2016) e algumas variáveis do andar não são responsivas ao medicamento, como a fase de balanço e a variabilidade do

andar (CURTZE et al., 2015; ROCHESTER et al., 2011). Desta forma, investigar o uso de diferentes intervenções para a reabilitação de pacientes com DP, como a ETCC, são necessárias.

A ETCC é uma técnica de estimulação cerebral não invasiva que envolve a aplicação de correntes elétricas de baixa intensidade em regiões de interesse do córtex cerebral, capaz de modular a excitabilidade cortical, mas insuficiente para gerar potencial de ação (NITSCHE et al., 2002, 2008). Um dispositivo de corrente contínua fornece correntes elétricas (geralmente de 1-2 mA) por meio de eletrodos anódicos (positivos) e catódicos (negativos) colocados em locais específicos no couro cabeludo (BRUNONI et al., 2012; NITSCHE et al., 2008). A estimulação anódica provoca o aumento da excitabilidade, enquanto a estimulação catódica diminui a excitabilidade das áreas alvos (NITSCHE et al., 2002) Entre as técnicas de estimulação cerebral, a ETCC se destaca pela segurança e facilidade no seu uso, pela possibilidade de aplicação clínica e pelo baixo custo quando comparado com outras técnicas de estimulação cerebral, como a estimulação magnética transcraniana. Embora a ETCC apresente resultados promissores, seus efeitos no andar de pacientes com DP ainda são controversos.

Uma meta-análise recente demonstrou que a ETCC gera efeito benéfico de curto prazo no andar de pacientes com DP, mas a magnitude deste efeito foi pequena (LEE et al., 2019). Alguns estudos demonstraram melhora no andar após a ETCC (DA SILVA et al., 2018; DAGAN et al., 2018; MISHRA; THRASHER, 2021), porém, outros não (BENNINGER et al., 2010; BUENO et al., 2019; DORUK et al., 2014). Possíveis explicações para estes achados são os diferentes protocolos de ETCC aplicados. Ainda, é crescente na literatura a aplicação da ETCC com diferentes intervenções concorrentes (motoras) para melhorar o andar (CONCEIÇÃO et al., 2021; YOTNUENGNIT et al., 2018) e os demais sintomas motores da DP (BERETTA et al., 2020). Neste sentido, uma revisão sistemática recente demonstrou que combinar a ETCC com outras formas de intervenção parece ocasionar mais benefícios no andar do que aplicar somente a ETCC (POL et al., 2021). Por exemplo, combinar ETCC anódica no

M1 com treinamento físico melhorou a velocidade do andar, mas este efeito não foi encontrado apenas com a aplicação da ETCC (KASKI et al., 2014b). Ainda, combinar sessões de tango com a ETCC anódica aplicada no M1 foi capaz de melhorar a resistência do andar (teste de 6 minutos) (KASKI et al., 2014a). Estimular o M1 se justifica pelo fato desta área estar envolvida na execução dos movimentos relacionados ao andar (PETERSEN et al., 2012). Outro estudo combinou a ETCC anódica aplicada no PFC com treino em ciclo ergômetro e encontrou melhora na fase de balanço, mas não verificou melhora em diversas outras variáveis do andar (velocidade, tempo em duplo suporte, comprimento do passo, etc.) (CONCEIÇÃO et al., 2021). O PFC é capaz de alocar recursos atencionais durante o andar e está envolvido na via locomotora indireta, que é mais acionada nos indivíduos com a via locomotora direta prejudicada, como os pacientes com DP (HEROLD et al., 2017). Por fim, outros estudos encontraram resultados similares entre as aplicações de ETCC sozinha e combinada com treinamento específico para o andar (COSTA-RIBEIRO et al., 2017) e fisioterapia (YOTNUENGNIT et al., 2018). Realizar a ETCC com treino físico parece ser uma estratégia promissora para melhorar o andar de pacientes com DP, mas ainda não há um consenso sobre qual é a área cortical ideal a ser estimulada e qual a melhor intervenção concorrente.

Baseado no exposto acima, o objetivo geral da pesquisa é verificar os efeitos agudos da ETCC anódica aplicada no M1 e no PFC combinada com treino em esteira no andar de pacientes com DP. O objetivo específico é comparar os efeitos entre a combinação da ETCC aplicada individualmente no M1 e no PFC com treino em esteira e a combinação da ETCC aplicada simultaneamente nestas regiões com treino em esteira nas variáveis do andar. O objetivo secundário é analisar a influência da combinação ETCC com treino em esteira na ativação do PFC em diferentes condições do andar, como o andar usual, com ultrapassagem de obstáculos e com tarefa dupla. Visto que estudos mostraram benefícios da ETCC (LEE et al., 2019) e do treinamento em esteira (MEHRHOLZ et al., 2015) no andar de pacientes, esperamos que a

combinação ETCC com treino em esteira potencialize a melhora na velocidade e na variabilidade do andar e que aumente a ativação do PFC durante o andar em diferentes condições.

3 MATERIAIS E MÉTODO

3.1 Participantes

O método de amostragem por conveniência será empregado, sendo que os pacientes com DP serão recrutados e selecionados do banco de dados do Programa de Atividade Física para Pacientes com doença de Parkinson (PROPARKI - UNESP Rio Claro). Análise a priori no software G*Power 3.1 foi realizada com base em um estudo anterior do nosso grupo (CONCEIÇÃO et al., 2021) inserindo os seguintes parâmetros: partial eta-squared = 0,27; poder estatístico = 0,80 e $\alpha < 0,05$. A variável e o teste estatístico utilizada no cálculo foram a velocidade do andar e ANOVA two-way para medidas repetidas (within factors), respectivamente. A análise indicou tamanho amostral total de 28 participantes, entretanto, 7 participantes já seriam o suficiente em virtude de o projeto possuir delineamento crossover. Com o intuito de prever perdas amostrais, 10 pacientes serão convidados a participar da pesquisa. Como critério de inclusão, os pacientes deverão ter o diagnóstico da DP idiopática dado por um neurologista particular, segundo os critérios do Banco de Cérebro de Londres (HUGHES et al., 1992). Os critérios de exclusão serão: i) possuir outra doença neurológica além da DP; ii) estar acima do estágio 3 na escala de Hoehn & Yahr (H&Y) adaptada (GOETZ et al., 2004); iii) possuir características que tornam a ETCC arriscada (implantes no neurais; histórico de convulsões; marca-passo; epilepsia); iv) histórico de problemas ortopédicos e/ou de visão que impossibilitem o cumprimento dos procedimentos experimentais e v) indicativo de demência (pontuação <20 no Mini-Exame do Estado Mental – MEEM) (BRUCKI et al., 2003).

3.2 Delineamento experimental

O presente projeto de pesquisa será um ensaio clínico randomizado, crossover, duplo-cego e sham-controlado e seguirá as recomendações do checklist CONSORT (Anexo A). Todos os procedimentos experimentais serão realizados nas dependências do LEPLO, da Universidade Estadual Paulista (Unesp), Instituto de Biociências, Rio Claro. O projeto será submetido ao Comitê de Ética em Pesquisa da mesma Universidade. Após aprovação do Comitê, o estudo será registrado na plataforma Registro Brasileiro de Ensaios Clínicos (ReBEC). Após fornecerem consentimento por meio da assinatura do Termo de Consentimento Livre e Esclarecido (Apêndice B), os pacientes serão convidados a virem ao LEPLO em cinco dias diferentes. No dia 1, os participantes farão avaliações clínicas, cognitivas, demográficas e familiarização com a esteira (observação da velocidade confortável na esteira para cada paciente). Nos dias 2, 3, 4 e 5, os participantes realizarão, sequencialmente, os seguintes procedimentos experimentais: i) avaliações pré-intervenção (andar e atividade cortical); ii) sessão de ETCC anódica (M1, PFC, M1+PFC ou sham) combinada com treino em esteira; e iii) avaliações pós-intervenção (andar e atividade cortical). O intervalo entre as sessões de intervenção será de uma semana para evitar que os efeitos ocasionados por uma sessão influenciem nas outras. Ainda, a sequência de exposição às sessões será contrabalanceada e randomizada entre os participantes em proporção de 1:1:1:1 por meio de um gerador de números aleatórios online (www.randomization.com). Para garantir a característica duplo-cego do estudo, os participantes não saberão qual condição de ETCC irão receber em cada dia de intervenção e o membro da equipe de pesquisa, responsável por manipular o equipamento da estimulação, não participará das avaliações (pré e pós). Todos os procedimentos experimentais serão realizados no mesmo horário para cada paciente e todos estarão no estado "ON" da medicação específica para DP, entre 45 e 60 minutos após a ingestão do medicamento.

3.3 Avaliações clínicas

Uma anamnese será aplicada em todos os participantes para coletar dados sobre idade, histórico de problemas ortopédicos e vestibulares, medicamentos em uso, tempo de diagnóstico da DP e outras informações relevantes. Dados antropométricos, como altura e massa corporal, serão coletados. O histórico de quedas dos últimos 12 meses será obtido do banco de dados do LEPLO. A dose diária de levodopa será calculada seguindo as recomendações de Tomlinson e colaboradores (2010). O medo de quedas e o *freezing* dos pacientes serão avaliados por meio do *Falls Eficacy Scale – International* (FES-I) (YARDLEY et al., 2005) e do *Freezing of Gait Questionnaire* (FOG-Q) (GILADI et al., 2009), respectivamente. A *Movement Disorders Society – Unified Parkinson's Disease Rating Scale* parte III (MDS-UPDRS III) (GOETZ et al., 2008) será aplicada para identificar o grau de acometimento motor da DP. Além disso, o hemicorpo mais afetado pela DP será determinado por meio de itens da MDS-UPDRS III (3.3b até 3.17d). O hemisfério cerebral mais afetado pela DP será inferido a partir da determinação do hemicorpo contralateral mais afetado. A escala de H&Y adaptada será utilizada para identificar o estágio evolutivo da doença (GOETZ et al., 2004). O MEEM será aplicado para rastrear a condição cognitiva global dos pacientes (BRUCKI et al., 2003). Como complemento ao MEEM, as partes A e B do *Trail Making Test* serão aplicadas para rastrear a flexibilidade mental, velocidade de processamento e função executiva dos pacientes (TOMBAUGH, 2004), sendo que a diferença entre as partes B e A será reportada.

3.4 Avaliação do andar

Inicialmente, avaliadores treinados irão posicionar uma touca, que contém o sistema da fNIRS, na cabeça do participante para registrar a atividade do PFC. O posicionamento da touca seguirá o sistema internacional 10/20 da eletroencefalografia (EEG), que consiste em posicionar o ponto de referência da touca (Cz) no ponto médio entre o násio e ínio e os pontos

pré-auriculares. Os optodos da fNIRS contidos na touca serão posicionados na parte frontal da cabeça, correspondente as áreas 9, 10 e 46 de Brodmann do hemisfério esquerdo e direito, que representam o PFC dorsolateral e anterior (ORCIOLI-SILVA et al., 2020, 2021a). Um acelerômetro será colocado na quinta vértebra lombar para obtenção das variáveis espaço-temporais do andar. A altura em que o acelerômetro estará posicionado na lombar será registrada. Após estas preparações, o participante andará em um circuito de 26,8m de comprimento em velocidade preferida nas seguintes condições: i) andar com velocidade usual; ii) andar com tarefa dupla cognitiva; e iii) andar com ultrapassagem de obstáculos. Os participantes realizarão 3 tentativas em cada uma das condições experimentais. Cada tentativa terá duração total de 60s. Inicialmente, o participante ficará em pé parado por 30s e será instruído a olhar para frente, permanecer em silêncio e realizar contagens simples mentalmente (adição de 1 em 1) para padronizar a demanda atencional durante o período baseline. (HOLTZER et al., 2015). Após um sinal verbal ("prepara, vai"), o participante realizará a condição experimental por 30s. Na condição com ultrapassagem de obstáculos, 4 obstáculos feitos de espuma (60cm de comprimento x 5cm de largura x 15cm de altura) estarão dispostos ao longo do circuito (ORCIOLI-SILVA et al., 2021b). Na condição com tarefa dupla cognitiva, um áudio, contendo números aleatórios de 1 a 9, tocará durante os 30s do andar e o participante deverá contar mentalmente quantas vezes uma determinada classe de números (pares ou ímpares) foi expressa (ORCIOLI-SILVA et al., 2020). O participante será instruído sobre qual classe de números deverá contar imediatamente antes do início do andar e dará sua resposta ao final da tentativa. A ordem das condições experimentais será randomizada para cada indivíduo. Os equipamentos (fNIRS e acelerômetro) serão removidos para realização do protocolo de intervenção, mas marcações serão realizadas para garantir o mesmo posicionamento dos equipamentos nas avaliações pós-intervenção.

3.5 Protocolo da ETCC combinada com treino em esteira

Após as avalições pré-intervenção, o participante ficará sentado em uma cadeira confortável para medição da pressão arterial (método auscultatório), da frequência cardíaca (FC) de repouso e para o preparo da ETCC. Eletrodos em esponjas embebidas com solução salina (35cm²), conectados ao estimulador MicroEstim Genius (NKL Produtos Eletrônicos Ltda. – EEP, Brusque/Santa Catarina, Brasil), serão colocados nas regiões alvos. Os participantes realizarão quatro dias de intervenção com a ETCC anódica: 1 dia no M1, 1 dia no PFC, 1 dia em ambas as áreas simultaneamente e 1 dia será sham/placebo. Nos quatro dias de intervenção, os eletrodos ânodos serão posicionados no M1 (posição C3 ou C4 do sistema 10-20) e no PFC (posição F3 ou F4 do sistema 10-20) do hemisfério cerebral mais afetado pela DP. O eletrodo cátodo (referência) será posicionado na região supraorbital (posição FP1 ou FP2 do sistema 10-20) do hemisfério contralateral aos eletrodos ânodos. Nos dias em que a estimulação for aplicada em apenas uma área, o eletrodo ânodo estará ligado somente no M1 ou no PFC. A montagem dos eletrodos foi pensada desta forma para que os pacientes e o membro da equipe responsável por administrar as sessões de intervenção não saibam qual é a condição de ETCC aplicada. Nas três sessões de ETCC ativa, a intensidade da corrente elétrica será de 2mA durante 20 min. Além deste período, haverá 30s iniciais de subida da corrente (até atingir 2mA) e mais 30s finais de descida da corrente. Na sessão de ETCC sham, haverá os 30s iniciais de subida, estimulação ativa (2mA) por 10s e os 30s finais de descida da corrente elétrica. O equipamento estará desligado nos 19min e 50s restantes.

O treino na esteira ergométrica ATL (Inbramed – Indústria Brasileira de Equipamentos Médicos Ltda., Porto Alegre/Rio Grande do Sul, Brasil) terá duração total de 30 min e a intensidade será controlada pela FC máxima (FCmax). O protocolo do treino envolverá: i) aquecimento de 5 min com FC entre 50-60% da FCmax; ii) parte principal de 20 min com intensidade entre 60-70% da FCmax e iii) volta à calma de 5 min com FC abaixo de 60% da

FCmax. A ETCC estará ligada (ativa ou *sham*) na parte principal do treino e desligada no aquecimento e na volta à calma. O avaliador irá aumentar e/ou diminuir a velocidade da esteira para manter a FC entre os limites estabelecidos. A FC será monitorada e registrada a cada minuto por meio do monitor cardíaco Polar V800 (Polar Electro Brasil Comércio, Distribuição, Importação e Exportação Ltda., São Paulo/SP, Brasil). A FCmax será estimada pela seguinte equação: FCmax = 208 – 0,7*idade) (TANAKA; MONAHAN; SEALS, 2001). Para os participantes que ingerem medicamentos betabloqueadores, a FCmax será estimada pela equação: FCmax = 164 – 0,7*idade) (BRAWNER et al., 2004). Ainda, a percepção subjetiva de esforço será monitorada a cada minuto por meio da escala de Borg modificada (0-10 índices) (BORG, 1982). Para segurança, um membro da equipe estará perto da esteira e o participante estará com um equipamento arnês durante todo o treino. Este equipamento é composto por um cinturão de segurança modelo CG 700/750, acoplado a um trava quedas retrátil modelo CG 500N (Carbografite Equipamentos Industriais Ltda., São Paulo/SP, Brasil), que estará preso a um sistema de polias fixado no teto. Imediatamente após as intervenções, os participantes serão questionados sobre sensações de desconforto e efeitos colaterais provocados pela ETCC (BRUNONI et al., 2011) e a pressão arterial será mensurada novamente.

3.6 Análise dos dados

3.6.1 Análise do andar

As variáveis do andar serão obtidas por meio da aceleração do centro de massa. Os dados do acelerômetro Trigno™ Avanti Sensor serão transmitidos via wi-fi para a estação de base do sistema Trigno™ Research+ (Delsys, Inc., Natick Massachusetts, EUA), e deste para um computador via cabo. O acelerômetro possui frequência de coleta de 148,15 Hz e seus dados serão armazenados no software EMGworks® (Delsys, Inc., Natick, Massachusetts, EUA). As variáveis do andar serão obtidas por meio de algoritmos em ambiente MATLAB® R2015a (The

MathWorks, Inc., Natick, Massachusetts, EUA), validados anteriormente por Del Din e colaboradores (2016). Resumidamente, os dados de aceleração serão transformados por meio de um sistema de coordenada vertical-horizontal e serão filtrados com filtro Butterworth de 4ª ordem (20 Hz) (MCCAMLEY et al., 2012; ZIJLSTRA; HOF, 2003). Os contatos iniciais e finais do ciclo do andar serão estimados por meio da *continuous wavelet transform* (CWT) da aceleração vertical, que será primeiro integrada e depois diferenciada usando um CWT Gaussiano. Os eventos de contato inicial e final serão detectados por meio dos pontos mínimos e máximos do CWT, respectivamente (DEL DIN; GODFREY; ROCHESTER, 2016). Ambos os contatos (pé-solo) do pé direito e esquerdo serão identificados. Os tempos de contato inicial e final serão usados para estimar a duração do passo e a fase de balanço (DEL DIN; GODFREY; ROCHESTER, 2016). O comprimento do passo será determinado a partir dos eventos de contato inicial por meio da aplicação do modelo de pêndulo invertido descrito por Zijlstra e Hof (2003). Velocidade e comprimento do passo, duração da fase de balanço, duração do duplo suporte e suas respectivas variabilidades (coeficiente de variação = desvio-padrão/média*100) serão analisadas. Na condição andar com ultrapassagem de obstáculos, os contatos com os obstáculos serão registados e removidos da análise. O desempenho da tarefa cognitiva durante o andar com tarefa dupla será quantificado pelo erro absoluto (resposta correta – resposta falada pelo participante). Todos os passos das três tentativas por condição serão considerados na análise.

3.6.2 Análise da atividade cortical

Os dados da atividade do PFC serão adquiridos por meio de um dispositivo fNIRS OctaMon portátil de 8 canais (Artinis Medical Systems, Elst, The Netherlands), com frequência de coleta de 10 Hz. A fNIRS é uma técnica de neuroimagem funcional não-invasiva que registra as mudanças na concentração de oxi-hemoglobina (HbO_2) e desoxi-hemoglobina. Apenas as

concentrações de HbO_2 serão analisadas pois este é um indicador mais sensível da atividade cortical durante o andar (SUZUKI et al., 2004). O modelo OctaMon contém 10 optodos, sendo 8 emissores de luz e 2 receptores de luz (4 emissores e 1 receptor por hemisfério), que emitem ondas de luz no comprimento de 760 a 850 mm. A distância entre os optodos é de 35 mm. O amplificador do sinal da fNIRS será posicionado na própria touca (região da nuca) e este sinal amplificado será enviado em tempo real, via *bluetooth* para um computador. O software OxySoft 3.0.52 (Artinis Medical Systems, Elst, The Netherlands) será utilizado para coleta, armazenamento e cálculo das concentrações de HbO_2 por meio da lei modificada de *Beer-Lambert*.

Os procedimentos de análise dos sinais da fNIRS (correções de artefatos e filtragem) serão realizados no software de fonte aberta NIRS-SPM (http://www.fil.ion.ucl.ac.uk/spm/software/) (YE et al., 2009) e seguirão as recomendações de Vitório e colaboradores (2017). Primeiramente, um filtro *wavelet-minimum description length* (*Wavelet* MDL) (BRIGADOI et al., 2014; COOPER et al., 2012; JANG et al., 2009) será utilizado para decompor o sinal em tendência global, resposta hemodinâmica e componentes de ruído. Ainda, ruídos de alta frequência serão reduzidos/removidos por meio de um filtro passa-baixa baseado na função canônica da resposta hemodinâmica (YE et al., 2009). Em seguida, algoritmos customizados escritos em ambiente MATLAB® R2015a (The MathWorks, Inc., Natick, Massachusetts, EUA), serão utilizados para os cálculos das variáveis de interesse. As concentrações de HbO_2 dos oito canais serão calculados, sendo quatro canais para cada hemisfério. Para análise dos dados, a tarefa será dividida em dois períodos: baseline (últimos 10 segundos antes do início do andar, quando o participante está na posição em pé parado) e andar (período entre o quinto e vigésimo quinto segundo após o início do andar). A média de concentração de HbO_2 de cada período analisado e para cada hemisfério cerebral (considerando a média de todas as tentativas realizadas por condição) será calculada. A diferença de

concentração de HbO_2 entre os períodos (andar – baseline) será calculada para avaliar a mudança relativa da atividade cortical entre o andar e o baseline (MAIDAN et al., 2016; MIRELMAN et al., 2014).

3.7 Análise estatística

O software SPSS® 22.0 (International Business Machines Corporation, Armonk, Nova Iorque, EUA) será utilizado para o tratamento estatístico e o nível de significância será mantido em < 0,05 para todas as análises. Os testes Shapiro-Wilk e de Levene serão aplicados para verificar a normalidade na distribuição dos dados e a homogeneidade das variâncias, respectivamente. Os dados com distribuição normal serão apresentados por meio de média ± desvio padrão enquanto os dados com distribuição não normal serão apresentados com mediana (quartis 25 e 75). Os deltas (Δ = pós – pré) das variáveis do andar e da concentração de HbO2 de cada sessão de intervenção serão calculados. Em seguida, ANOVAs two-way com medidas repetidas para os fatores condição do andar (usual X com ultrapassagem de obstáculos X tarefa dupla cognitiva) e condição de estimulação (M1 X PFC X M1+PFC X sham) serão aplicadas. Em caso de interação entre os fatores, testes post hoc de Bonferroni serão empregados para identificar as diferenças. A média de resposta padronizada (MRP) será utilizada para verificar a magnitude da intervenção nas variáveis do andar. Valores de MRP serão interpretados como trivial (<0,2), baixo (≥0,2), moderado (≥0,5) e alto (≥0,8) (MIDDEL; VAN SONDEREN, 2002).

REFERÊNCIAS BIBLIOGRÁFICAS

ASCHERIO, Alberto; SCHWARZSCHILD, Michael A. The epidemiology of Parkinson's disease: risk factors and prevention. **The Lancet Neurology**, London, v. 15, n. 12, p. 1257–1272, 2016. DOI: 10.1016/S1474-4422(16)30230-7.

BARBOSA, Maira Tonidandel; CARAMELLI, Paulo; MAIA, Débora Palma; CUNNINGHAM, Mauro César Quintão; GUERRA, Henrique Leonardo; LIMA-COSTA, Maria Fernanda; CARDOSO, Francisco. Parkinsonism and Parkinson's disease in the elderly: A community-based survey in Brazil (the Bambuí study). **Movement Disorders**, New York, v. 21, n. 6, p. 800–808, 2006. DOI: 10.1002/mds.20806.

BENNINGER, David H.; LOMAREV, Mikhail; LOPEZ, Grisel; WASSERMANN, Eric M.; LI, Xiaobai; CONSIDINE, Elaine; HALLETT, Mark. Transcranial direct current stimulation for the treatment of Parkinson's disease. **Journal of Neurology, Neurosurgery & Psychiatry**, London, v. 81, n. 10, p. 1105–1111, 2010. DOI: 10.1136/jnnp.2009.202556.

BERETTA, Victor Spiandor; CONCEIÇÃO, Núbia Ribeiro; NÓBREGA-SOUSA, Priscila; ORCIOLI-SILVA, Diego; DANTAS, Luana Karla Braz Fonseca; GOBBI, Lilian Teresa Bucken; VITÓRIO, Rodrigo. Transcranial direct current stimulation combined with physical or cognitive training in people with Parkinson's disease: a systematic review. **Journal of NeuroEngineering and Rehabilitation**, London, v. 17, n. 1, p. 74, 2020. DOI: 10.1186/s12984-020-00701-6.

BORG, G. A. Psychophysical bases of perceived exertion. **Medicine and science in sports and exercise**, *[S. l.]*, v. 14, n. 5, p. 377–81, 1982.

BRAWNER, Clinton A.; EHRMAN, Jonathan K.; SCHAIRER, John R.; CAO, Jie J.; KETEYIAN, Steven J. Predicting maximum heart rate among patients with coronary heart disease receiving beta-adrenergic blockade therapy. **American heart journal**, *[S. l.]*, v. 148, n. 5, p. 910–914, 2004. DOI: 10.1016/J.AHJ.2004.04.035.

BRIGADOI, Sabrina; CECCHERINI, Lisa; CUTINI, Simone; SCARPA, Fabio; SCATTURIN, Pietro; SELB, Juliette; GAGNON, Louis; BOAS, David A.; COOPER, Robert J. Motion artifacts in functional near-infrared spectroscopy: a comparison of motion correction techniques applied to real cognitive data. **NeuroImage**, *[S. l.]*, v. 85 Pt 1, n. 0 1, p. 181–191, 2014. DOI: 10.1016/J.NEUROIMAGE.2013.04.082.

BRUCKI, Sonia M. D.; NITRINI, Ricardo; CARAMELLI, Paulo; BERTOLUCCI, Paulo H. F.; OKAMOTO, Ivan H. Sugestões para o uso do mini-exame do estado mental no Brasil. **Arquivos de Neuro-Psiquiatria**, *[S. l.]*, v. 61, n. 3B, p. 777–781, 2003. DOI: 10.1590/S0004-282X2003000500014.

BRUNONI, Andre Russowsky et al. Clinical research with transcranial direct current stimulation (tDCS): Challenges and future directions. **Brain Stimulation**, *[S. l.]*, v. 5, n. 3, p. 175–195, 2012. DOI: 10.1016/j.brs.2011.03.002.

BRUNONI, Andre Russowsky; AMADERA, Joao; BERBEL, Bruna; VOLZ, Magdalena Sarah; RIZZERIO, Brenno Gomes; FREGNI, Felipe. A systematic review on reporting and assessment of adverse effects associated with transcranial direct current stimulation. **International Journal of Neuropsychopharmacology**, *[S. l.]*, v. 14, n. 8, p. 1133–1145, 2011. DOI: 10.1017/S1461145710001690.

BUENO, Maria Eduarda Brandão; DO NASCIMENTO NETO, Luiz Inácio; TERRA, Marcelle Brandão; BARBOZA, Natália Mariano; OKANO, Alexandre Hideki; SMAILI, Suhaila Mahmoud. Effectiveness of acute transcranial direct current stimulation on non-motor and motor symptoms in Parkinson's disease. **Neuroscience Letters**, *[S. l.]*, v. 696, p. 46–51, 2019. DOI: 10.1016/j.neulet.2018.12.017.

CONCEIÇÃO, Núbia Ribeiro; GOBBI, Lilian Teresa Bucken; NÓBREGA-SOUSA, Priscila; ORCIOLI-SILVA, Diego; BERETTA, Victor Spiandor; LIRANI-SILVA, Ellen; OKANO, Alexandre Hideki; VITÓRIO, Rodrigo. Aerobic Exercise Combined With Transcranial Direct Current Stimulation Over the Prefrontal Cortex in Parkinson Disease: Effects on Cortical Activity, Gait, and Cognition. **Neurorehabilitation and Neural Repair**, *[S. l.]*, v. 35, n. 8, p. 717–728, 2021. DOI: 10.1177/15459683211019344.

COOPER, Robert J.; SELB, Juliette; GAGNON, Louis; PHILLIP, Dorte; SCHYTZ, Henrik W.; IVERSEN, Helle K.; ASHINA, Messoud; BOAS, David A. A systematic comparison of motion artifact correction techniques for functional near-infrared spectroscopy. **Frontiers in neuroscience**, *[S. l.]*, v. 6, n. OCT, 2012. DOI: 10.3389/FNINS.2012.00147.

COSTA-RIBEIRO, Adriana et al. Transcranial direct current stimulation associated with gait training in Parkinson's disease: A pilot randomized clinical trial. **Developmental Neurorehabilitation**, *[S. l.]*, v. 20, n. 3, p. 121–128, 2017. DOI: 10.3109/17518423.2015.1131755.

CURTZE, Carolin; NUTT, John G.; CARLSON-KUHTA, Patricia; MANCINI, Martina; HORAK, Fay B. Levodopa Is a Double-Edged Sword for Balance and Gait in People With Parkinson's Disease. **Movement Disorders**, New York, v. 30, n. 10, p. 1361–1370, 2015. DOI: 10.1002/mds.26269.

DA SILVA, Débora Cristina Lima; LEMOS, Thiago; DE SÁ FERREIRA, Arthur; HORSCZARUK, Carlos Henrique Ramos; PEDRON, Carla Andressa; DE CARVALHO RODRIGUES, Erika; DE OLIVEIRA, Laura Alice Santos. Effects of Acute Transcranial Direct Current Stimulation on Gait Kinematics of Individuals With Parkinson Disease. **Topics in Geriatric Rehabilitation**, *[S. l.]*, v. 34, n. 4, p. 262–268, 2018. DOI: 10.1097/TGR.0000000000000203.

DAGAN, Moria; HERMAN, Talia; HARRISON, Rachel; ZHOU, Junhong; GILADI, Nir; RUFFINI, Giulio; MANOR, Brad; HAUSDORFF, Jeffrey M. Multitarget transcranial direct current stimulation for freezing of gait in Parkinson's disease. **Movement Disorders**, *[S. l.]*, v. 33, n. 4, p. 642–646, 2018. DOI: 10.1002/mds.27300.

DEL DIN, Silvia; GODFREY, Alan; ROCHESTER, Lynn. Validation of an Accelerometer to Quantify a Comprehensive Battery of Gait Characteristics in Healthy Older Adults and Parkinson's Disease: Toward Clinical and at Home Use. **IEEE Journal of Biomedical and Health Informatics**, *[S. l.]*, v. 20, n. 3, p. 838–847, 2016. DOI: 10.1109/JBHI.2015.2419317.

DORUK, Deniz; GRAY, Zachary; BRAVO, Gabriela L.; PASCUAL-LEONE, Alvaro; FREGNI, Felipe. Effects of tDCS on executive function in Parkinson's disease. **Neuroscience Letters**, *[S. l.]*, v. 582, p. 27–31, 2014. DOI: 10.1016/j.neulet.2014.08.043.

FREGNI, Felipe et al. Noninvasive cortical stimulation with transcranial direct current stimulation in Parkinson's disease. **Movement Disorders**, New York, v. 21, n. 10, p. 1693–1702, 2006. DOI: 10.1002/mds.21012.

GALNA, Brook; MURPHY, Anna T.; MORRIS, Meg E. Obstacle crossing in people with Parkinson's disease: Foot clearance and spatiotemporal deficits. **Human Movement Science**, *[S. l.]*, v. 29, n. 5, p. 843–852, 2010. DOI: 10.1016/j.humov.2009.09.006.

GALVAN, Adriana; WICHMANN, Thomas. Pathophysiology of parkinsonism. **Clinical neurophysiology : official journal of the International Federation of Clinical Neurophysiology**, *[S. l.]*, v. 119, n. 7, p. 1459–1474, 2008. DOI: 10.1016/J.CLINPH.2008.03.017.

GBD 2016 PARKINSON'S DISEASE COLLABORATORS. Global, regional, and national burden of Parkinson's disease, 1990–2016: a systematic analysis for the Global Burden of Disease Study 2016. **The Lancet Neurology**, London, v. 17, n. 11, p. 939–953, 2018. DOI: 10.1016/S1474-4422(18)30295-3.

GILADI, Nir et al. Validation of the freezing of gait questionnaire in patients with Parkinson's disease. **Movement Disorders**, New York, v. 24, n. 5, p. 655–661, 2009. DOI: 10.1002/mds.21745.

GOETZ, Christopher G. et al. Movement Disorder Society Task Force report on the Hoehn and Yahr staging scale: Status and recommendations The Movement Disorder Society Task Force on rating scales for Parkinson's disease. **Movement Disorders**, New York, v. 19, n. 9, p. 1020–1028, 2004. DOI: 10.1002/mds.20213.

GOETZ, Christopher G. et al. Movement Disorder Society-sponsored revision of the Unified Parkinson's Disease Rating Scale (MDS-UPDRS): Scale presentation and clinimetric testing results. **Movement Disorders**, New York, v. 23, n. 15, p. 2129–2170, 2008. DOI: 10.1002/mds.22340.

HEROLD, Fabian; WIEGEL, Patrick; SCHOLKMANN, Felix; THIERS, Angelina; HAMACHER, Dennis; SCHEGA, Lutz. Functional near-infrared spectroscopy in movement science: a systematic review on cortical activity in postural and walking tasks. **Neurophotonics**, *[S. l.]*, v. 4, n. 4, p. 041403, 2017. DOI: 10.1117/1.NPh.4.4.041403.

HOLTZER, Roee; MAHONEY, Jeannette R.; IZZETOGLU, Meltem; WANG, Cuiling; ENGLAND, Sarah; VERGHESE, Joe. Online fronto-cortical control of simple and attention-demanding locomotion in humans. **NeuroImage**, *[S. l.]*, v. 112, p. 152–159, 2015. DOI: 10.1016/j.neuroimage.2015.03.002.

HUGHES, Andrew J.; DANIEL, Susan E.; KILFORD, Linda; LEES, Andrew J. Accuracy of clinical diagnosis of idiopathic Parkinson's disease: a clinico-pathological study of 100 cases. **Journal of Neurology, Neurosurgery & Psychiatry**, London, v. 55, n. 3, p. 181–184, 1992. DOI: 10.1136/jnnp.55.3.181.

JANG, Kwang-Eun; TAK, Sungho; JUNG, Jinwook; JANG, Jaeduck; JEONG, Yong; YE, Yong Chul. Wavelet minimum description length detrending for near-infrared spectroscopy. **https://doi.org/10.1117/1.3127204**, *[S. l.]*, v. 14, n. 3, p. 034004, 2009. DOI: 10.1117/1.3127204.

JANKOVIC, Joseph. Parkinson's disease therapy: tailoring choices for early and late disease, young and old patients. **Clinical Neuropharmacology**, *[S. l.]*, v. 23, n. 5, p. 252–61, 2000. DOI: 10.1097/00002826-200009000-00003.

KASKI, D.; ALLUM, J. H.; BRONSTEIN, A. M.; DOMINGUEZ, R. O. Applying anodal tDCS during tango dancing in a patient with Parkinson's disease. **Neuroscience Letters**, *[S. l.]*, v. 568, p. 39–43, 2014. a. DOI: 10.1016/j.neulet.2014.03.043.

KASKI, D.; DOMINGUEZ, RO; ALLUM, JH; ISLAM, AF; BRONSTEIN, AM. Combining physical training with transcranial direct current stimulation to improve gait in Parkinson's disease: a pilot randomized controlled study. **Clinical Rehabilitation**, *[S. l.]*, v. 28, n. 11, p. 1115–1124, 2014. b. DOI: 10.1177/0269215514534277.

KELLY, Valerie E.; EUSTERBROCK, Alexis J.; SHUMWAY-COOK, Anne. A Review of Dual-Task Walking Deficits in People with Parkinson's Disease: Motor and Cognitive Contributions, Mechanisms, and Clinical Implications. **Parkinson's Disease**, *[S. l.]*, v. 2012, p. 1–14, 2012. DOI: 10.1155/2012/918719.

LEE, Hyo Keun; AHN, Se Ji; SHIN, Yang Mi; KANG, Nyeonju; CAURAUGH, James H. Does transcranial direct current stimulation improve functional locomotion in people with Parkinson's disease? A systematic review and meta-analysis. **Journal of NeuroEngineering and Rehabilitation**, *[S. l.]*, v. 16, n. 1, p. 84, 2019. DOI: 10.1186/s12984-019-0562-4.

MAIDAN, Inbal et al. The role of the frontal lobe in complex walking among patients With Parkinson's Disease and healthy older adults: An fNIRS study. **Neurorehabilitation and Neural Repair**, New York, v. 30, n. 10, p. 963–971, 2016. DOI: 10.1177/1545968316650426.

MCCAMLEY, John; DONATI, Marco; GRIMPAMPI, Eleni; MAZZÀ, Claudia. An enhanced estimate of initial contact and final contact instants of time using lower trunk inertial sensor data. **Gait & Posture**, *[S. l.]*, v. 36, n. 2, p. 316–318, 2012. DOI: 10.1016/j.gaitpost.2012.02.019.

MEHRHOLZ, Jan; KUGLER, Joachim; STORCH, Alexander; POHL, Marcus; HIRSCH, Kathleen; ELSNER, Bernhard. Treadmill training for patients with Parkinson's disease. **Cochrane Database of Systematic Reviews**, *[S. l.]*, v. 2015, n. 9, 2015. DOI: 10.1002/14651858.CD007830.pub4.

MIDDEL, Berrie; VAN SONDEREN, Eric. Statistical significant change versus relevant or important change in (quasi) experimental design: some conceptual and methodological problems in estimating magnitude of intervention-related change in health services research. **International Journal of Integrated Care**, *[S. l.]*, v. 2, n. 4, 2002. DOI: 10.5334/ijic.65.

MIRELMAN, Anat et al. Gait impairments in Parkinson's disease. **The Lancet Neurology**, London, v. 18, n. 7, p. 697–708, 2019. DOI: 10.1016/S1474-4422(19)30044-4.

MIRELMAN, Anat; MAIDAN, Inbal; BERNAD-ELAZARI, Hagar; NIEUWHOF, Freek; REELICK, Miriam; GILADI, Nir; HAUSDORFF, M. Jeffrey. Increased frontal brain activation during walking while dual tasking: An fNIRS study in healthy young adults. **Journal of NeuroEngineering and Rehabilitation**, *[S. l.]*, v. 11, n. 1, p. 1–7, 2014. DOI: 10.1186/1743-0003-11-85.

MISHRA, Ram kinker; THRASHER, Adam T. Transcranial direct current stimulation of dorsolateral prefrontal cortex improves dual-task gait performance in patients with Parkinson's disease: A double blind, sham-controlled study. **Gait & Posture**, *[S. l.]*, v. 84, p. 11–16, 2021. DOI: 10.1016/j.gaitpost.2020.11.012.

NIEUWHOF, Freek; REELICK, Miriam F.; MAIDAN, Inbal; MIRELMAN, Anat; HAUSDORFF, Jeffrey M.; OLDE RIKKERT, Marcel G. M.; BLOEM, Bastiaan R.; MUTHALIB, Makii; CLAASSEN, Jurgen A. H. R. Measuring prefrontal cortical activity during dual task walking in patients with Parkinson's disease: feasibility of using a new portable fNIRS device. **Pilot and Feasibility Studies**, *[S. l.]*, v. 2, n. 1, p. 59, 2016. DOI: 10.1186/s40814-016-0099-2.

NITSCHE, M. A.; LIEBETANZ, D.; TERGAU, F.; PAULUS, W. Modulation of cortical excitability by transcranial direct current stimulation. **Der Nervenarzt**, *[S. l.]*, v. 73, n. 4, p. 332–335, 2002. DOI: 10.1007/s00115-002-1272-9.

NITSCHE, Michael A. et al. Transcranial direct current stimulation: State of the art 2008. **Brain Stimulation**, *[S. l.]*, v. 1, n. 3, p. 206–223, 2008. DOI: 10.1016/j.brs.2008.06.004.

NONNEKES, Jorik; TIMMER, Monique H. M.; DE VRIES, Nienke M.; RASCOL, Olivier; HELMICH, Rick C.; BLOEM, Bastiaan R. Unmasking levodopa resistance in Parkinson's disease. **Movement Disorders**, *[S. l.]*, v. 31, n. 11, p. 1602–1609, 2016. DOI: 10.1002/mds.26712.

ORCIOLI-SILVA, Diego; VITÓRIO, Rodrigo; BERETTA, Victor Spiandor; DA CONCEIÇÃO, Núbia Ribeiro; NÓBREGA-SOUSA, Priscila; OLIVEIRA, Anderson Souza; GOBBI, Lilian Teresa Bucken. Is Cortical Activation During Walking Different Between Parkinson's Disease Motor Subtypes? **The Journals of Gerontology: Series A**, *[S. l.]*, v. 76, n. 4, p. 561–567, 2021. a. DOI: 10.1093/gerona/glaa174.

ORCIOLI-SILVA, Diego; VITÓRIO, Rodrigo; NÓBREGA-SOUSA, Priscila; BERETTA, Victor Spiandor; CONCEIÇÃO, Núbia Ribeiro Da; OLIVEIRA, Anderson Souza; PEREIRA, Marcelo Pinto; GOBBI, Lilian Teresa Bucken. Cortical Activity Underlying Gait

Improvements Achieved With Dopaminergic Medication During Usual Walking and Obstacle Avoidance in Parkinson Disease. **Neurorehabilitation and Neural Repair**, *[S. l.]*, v. 35, n. 5, p. 406–418, 2021. b. DOI: 10.1177/15459683211000736.

ORCIOLI-SILVA, Diego; VITÓRIO, Rodrigo; NÓBREGA-SOUSA, Priscila; DA CONCEIÇÃO, Núbia Ribeiro; BERETTA, Victor Spiandor; LIRANI-SILVA, Ellen; GOBBI, Lilian Teresa Bucken. Levodopa Facilitates Prefrontal Cortex Activation During Dual Task Walking in Parkinson Disease. **Neurorehabilitation and Neural Repair**, *[S. l.]*, v. 34, n. 7, p. 589–599, 2020. DOI: 10.1177/1545968320924430.

PETERSEN, T. H.; WILLERSLEV-OLSEN, M.; CONWAY, B. A.; NIELSEN, J. B. The motor cortex drives the muscles during walking in human subjects. **The Journal of Physiology**, *[S. l.]*, v. 590, n. 10, p. 2443–2452, 2012. DOI: 10.1113/jphysiol.2012.227397. Disponível em: http://doi.wiley.com/10.1113/jphysiol.2012.227397.

PETERSON, D. S.; HORAK, F. B. Neural Control of Walking in People with Parkinsonism. **Physiology**, *[S. l.]*, v. 31, n. 2, p. 95–107, 2016. DOI: 10.1152/physiol.00034.2015.

POL, Fateme; SALEHINEJAD, Mohammad Ali; BAHARLOUEI, Hamzeh; NITSCHE, Michael A. The effects of transcranial direct current stimulation on gait in patients with Parkinson's disease: a systematic review. **Translational Neurodegeneration**, *[S. l.]*, v. 10, n. 1, p. 22, 2021. DOI: 10.1186/s40035-021-00245-2.

ROCHESTER, Lynn; BAKER, Katherine; NIEUWBOER, Alice; BURN, David. Targeting dopa-sensitive and dopa-resistant gait dysfunction in Parkinson's disease: Selective responses to internal and external cues. **Movement Disorders**, *[S. l.]*, v. 26, n. 3, p. 430–435, 2011. DOI: 10.1002/mds.23450.

STUART, Samuel; BELLUSCIO, Valeria; QUINN, Joseph F.; MANCINI, Martina. Pre-frontal Cortical Activity During Walking and Turning Is Reliable and Differentiates Across Young, Older Adults and People With Parkinson's Disease. **Frontiers in Neurology**, *[S. l.]*, v. 10, n. MAY, 2019. DOI: 10.3389/fneur.2019.00536.

SUZUKI, Mitsuo; MIYAI, Ichiro; ONO, Takeshi; ODA, Ichiro; KONISHI, Ikuo; KOCHIYAMA, Takanori; KUBOTA, Kisou. Prefrontal and premotor cortices are involved in adapting walking and running speed on the treadmill: an optical imaging study. **NeuroImage**, *[S. l.]*, v. 23, n. 3, p. 1020–6, 2004. DOI: 10.1016/j.neuroimage.2004.07.002.

TAKAKUSAKI, Kaoru; TOMITA, Nozomi; YANO, Masafumi. Substrates for normal gait and pathophysiology of gait disturbances with respect to the basal ganglia dysfunction. **Journal of Neurology**, *[S. l.]*, v. 255, n. S4, p. 19–29, 2008. DOI: 10.1007/s00415-008-4004-7

TANAKA, Hirofumi; MONAHAN, Kevin D.; SEALS, Douglas R. Age-predicted maximal heart rate revisited. **Journal of the American College of Cardiology**, *[S. l.]*, v. 37, n. 1, p. 153–156, 2001. DOI: 10.1016/S0735-1097(00)01054-8.

TARAZI, F. I.; SAHLI, Z. T.; WOLNY, M.; MOUSA, S. A. Emerging therapies for Parkinson's disease: from bench to bedside. **Pharmacology & therapeutics**, *[S. l.]*, v. 144, n. 2, p. 123–33, 2014. DOI: 10.1016/j.pharmthera.2014.05.010.

TOMBAUGH, T. Trail Making Test A and B: Normative data stratified by age and education. **Archives of Clinical Neuropsychology**, *[S. l.]*, v. 19, n. 2, p. 203–214, 2004. DOI: 10.1016/S0887-6177(03)00039-8.

TOMLINSON, Claire L.; STOWE, Rebecca; PATEL, Smitaa; RICK, Caroline; GRAY, Richard; CLARKE, Carl E. Systematic review of levodopa dose equivalency reporting in Parkinson's disease. **Movement Disorders**, New York, v. 25, n. 15, p. 2649–2653, 2010. DOI: 10.1002/mds.23429.

VITORIO, R.; STUART, S.; ROCHESTER, L.; ALCOCK, L.; PANTALL, A. fNIRS response during walking — Artefact or cortical activity? A systematic review. **Neuroscience & Biobehavioral Reviews**, *[S. l.]*, v. 83, p. 160–172, 2017. DOI: 10.1016/j.neubiorev.2017.10.002.

VITÓRIO, Rodrigo; LIRANI-SILVA, Ellen; BAPTISTA, André Macari; BARBIERI, Fabio Augusto; DOS SANTOS, Paulo Cezar Rocha; TEIXEIRA-ARROYO, Claudia; GOBBI, Lilian Teresa Bucken. Disease severity affects obstacle crossing in people with Parkinson's disease. **Gait & Posture**, *[S. l.]*, v. 40, n. 1, p. 266–269, 2014. DOI: 10.1016/j.gaitpost.2014.03.003.

VITÓRIO, Rodrigo; PIERUCCINI-FARIA, Frederico; STELLA, Florindo; GOBBI, Sebastião; GOBBI, Lilian Teresa Bucken. Effects of obstacle height on obstacle crossing in mild Parkinson's disease. **Gait & Posture**, *[S. l.]*, v. 31, n. 1, p. 143–146, 2010. DOI: 10.1016/j.gaitpost.2009.09.011.

WICHMANN, Thomas; DELONG, Mahlon R. Anatomy and physiology of the basal ganglia: relevance to Parkinson's disease and related disorders. *In*: **Handbook of clinical neurology**. [s.l.] : Handb Clin Neurol, 2007. v. 83p. 1–18. DOI: 10.1016/S0072-9752(07)83001-6.

YARDLEY, Lucy; BEYER, Nina; HAUER, Klaus; KEMPEN, Gertrudis; PIOT-ZIEGLER, Chantal; TODD, Chris. Development and initial validation of the Falls Efficacy Scale-International (FES-I). **Age and Ageing**, London, v. 34, n. 6, p. 614–619, 2005. DOI: 10.1093/ageing/afi196.

YE, Jong Chul; TAK, Sungho; JANG, Kwang Eun; JUNG, Jinwook; JANG, Jaeduck. NIRS-SPM: statistical parametric mapping for near-infrared spectroscopy. **NeuroImage**, *[S. l.]*, v. 44, n. 2, p. 428–447, 2009. DOI: 10.1016/J.NEUROIMAGE.2008.08.036.

YOTNUENGNIT, Pattarapol; BHIDAYASIRI, Roongroj; DONKHAN, Rattana; CHALUAYSRIMUANG, Juthamas; PIRAVEJ, Krisna. Effects of Transcranial Direct Current Stimulation Plus Physical Therapy on Gait in Patients With Parkinson Disease. **American Journal of Physical Medicine & Rehabilitation**, *[S. l.]*, v. 97, n. 1, p. 7–15, 2018. DOI: 10.1097/PHM.0000000000000783.

ZIJLSTRA, Wiebren; HOF, At L. Assessment of spatio-temporal gait parameters from trunk accelerations during human walking. **Gait & Posture**, *[S. l.]*, v. 18, n. 2, p. 1–10, 2003. DOI: 10.1016/S0966-6362(02)00190-X.
