# Supplementary material for: Aerobic exercise on the treadmill combined with transcranial direct current stimulation on the gait of people with Parkinson’s disease: A protocol for a randomized clinical trial
Source: PLoS One. 2024 Apr 25;19(4):e0300243. doi: 10.1371/journal.pone.0300243 (PMC11045059; doi:10.1371/journal.pone.0300243)
Supplement: S3 File — (PDF) [file pone.0300243.s004.pdf]

FREE AND INFORMED CONSENT TERMS
(National Health Council, Resolution 466/12)

I, Gabriel Antonio Gazziero Moraca, ID: 56.851.201-7, a master's student in the Interunits Graduate Program in Movement Sciences and the researcher leading this study, extend an invitation for you to participate as a volunteer in the research project titled "Effects of transcranial direct current stimulation combined with treadmill training on the gait of people with Parkinson's disease: a randomized clinical trial." The research team comprises Prof. Dr. Lilian Teresa Bucken Gobbi, Prof. Dr. Diego Orcioli-Silva, Prof. Dr. Victor Spiandor Beretta, and undergraduate student Beatriz Regina Legutke. All experimental procedures will be conducted at the Posture and Locomotion Studies Laboratory (LEPLO), within the Department of Physical Education at the Institute of Biosciences, São Paulo State University, Rio Claro (UNESP/RC).

The primary goal of this research is to evaluate the immediate effects of anodic transcranial direct current stimulation (tDCS) applied to the primary motor cortex and the prefrontal cortex, in conjunction with treadmill training, on the walking performance of patients with Parkinson's disease. The secondary objective is to examine the impact of integrating tDCS with treadmill training on prefrontal cortex activity in various walking scenarios, including regular walking, walking with obstacle avoidance, and walking with a cognitive dual task.

If you choose to participate in this research, you will be invited to attend LEPLO for five days, with a one-week interval between the last four days. On day 1, you will complete specific questionnaires addressing health issues, current medications, cognitive status, the extent of Parkinson's disease manifestation, fear of falls, and freezing of gait. Should you desire, your companion may accompany you during these assessments. Additionally, on day 1, your height and weight will be measured, and you will be requested to walk on a treadmill to determine the speed at which you feel comfortable. During days 2, 3, 4, and 5, you will engage in walking and cortical activity assessments, along with treadmill training combined with tDCS. The entire protocol, encompassing assessments and training, is anticipated to take approximately 1 hour and 10 minutes each day. Below, we provide a description of the procedures to enhance your understanding:

Gait and cortical assessments: A trained evaluator will make markings on your head to position a cap that records cortical activity. The contact of the cap is superficial and does not cause pain or discomfort. The assessor will attach an accelerometer to your back to assess your walking. Following these preparations, you will walk at your regular pace in a rectangular circuit under three conditions: usual walking, walking with obstacle avoidance, and walking with a cognitive dual task. You will perform three trials in each condition, and each trial will last a total of 1 minute. During the initial 30 seconds, you will stand still, look forward, remain silent, and mentally perform simple counting (1 + 1 + 1...). Upon the assessor's cue of "ready, go," you will walk for the next 30 seconds in the circuit. In the obstacle avoidance condition, four 15 cm high obstacles will be placed in the circuit. In the cognitive dual task condition, an audio will play numbers from 1 to 9, and you should mentally count how many times you hear specific numbers. At the trial's conclusion, you should articulate your response. All these procedures will occur both before and after treadmill training combined with tDCS. At the conclusion of the pre-training assessments, the assessor will remove the equipment, but the markings will be retained to ensure the same equipment positioning in the post-training assessments.

Treadmill training combined of tDCS: Before commencing the training, you will be seated for the measurement of your blood pressure and heart rate. Subsequently, the evaluator will place three moist sponge electrodes, secured with elastic bands, on your head for the application of stimulation. The stimulation poses no risk to you due to the low intensity of the electrical current, but there may be a slight tingling and/or itching sensation in the stimulated area during the initial seconds of application. The stimulation will last a total of 20 minutes and will be administered while you walk on the treadmill. The treadmill training protocol will endure for a total of 30 minutes, encompassing a warm-up (5 minutes without stimulation), the main part (20 minutes with stimulation), and cool down (5 minutes without stimulation). The evaluator will regulate the treadmill velocity and monitor your heart rate throughout the training. Finally, you will be required to complete a questionnaire regarding the sensations induced by the stimulation, and your blood pressure will be measured once again.

Despite the procedures being highly secure, there are potential risks of falls, discomfort, and embarrassment during your participation in the research. To mitigate these risks, all assessments and training sessions will be conducted by experienced and trained professionals. During walking and cortical activity assessments (pre- and post-training), an assessor will be present to assist in case of unexpected events. The obstacles used are made of foam with contrasting colors, reducing the risk of accidents and aiding in identification. While undergoing treadmill training with tDCS, you may experience tingling, burning, or irritation in the stimulated region, typically lasting only a few seconds at the beginning of stimulation. A team member will prompt you to report any enduring discomfort to ensure appropriate safety measures. Additionally, throughout the training, you will be secured with a safety harness attached to the ceiling to prevent falls. In the event of necessity, team members will provide first aid, and you will be directed to the nearest health center.

Your participation in this research will not yield immediate health benefits; nevertheless, the study's results may serve multiple purposes. The research holds the potential to enhance our understanding of the effects of a non-invasive stimulation technique combined with physical exercise on walking in diverse situations and on the cortical activity of individuals with Parkinson's disease. Furthermore, this research may play a role in developing novel rehabilitation procedures aimed at alleviating the motor impairments experienced by this population.

You will be assigned an identification code to maintain your anonymity throughout the research, and you are encouraged to seek clarification on the procedures whenever needed. Participation in the research is entirely voluntary, allowing you to decline or withdraw from the study at any time without incurring any financial or personal detriment. Additionally, it's important to emphasize that you will not receive any compensation for your participation, and there will be no expenses incurred on your part. All study results will be exclusively used for educational and research purposes. You have the right to request information about the test results at any time and can contact the Research Ethics Committee of the Institute of Biosciences at UNESP/RC for any clarifications regarding the research.

Your personal data, which includes identifying information such as name, ID, phone number, address, and date of birth, will never be shared and will be maintained in absolute confidentiality. I invite you to respond to the questions below concerning the reuse and sharing of your non-identifiable data (data that does not permit the detection of your identity). Please be aware that you have the option to withdraw your consent for the reuse and sharing of non-identifiable data at any time.

Do you allow your collected non-identifiable data to be stored and preserved in data repositories, such as the Institutional Repository of UNESP?

☐ Yes ☐ No

Do you allow your collected non-identifiable data to be published in specific scientific journals on research data?

☐ Yes ☐ No

Do you allow your collected non-identifiable data to be used in other studies by researchers from LEPLO?

☐ Yes ☐ No

Do you allow your collected non-identifiable data to be used in other studies by researchers from other institutions?

☐ Yes ☐ No

If you feel sufficiently informed about this research, its objectives, potential risks, and benefits, I invite you to sign this form, prepared in duplicate, with one copy for you and another for the responsible researcher.

Rio Claro, ______________________

________________________________ ________________________________

Research participant signature Researcher’s signature

Research Data:

Project title: Effects of transcranial direct current stimulation combined with treadmill training on the gait of people with Parkinson's disease: a randomized clinical trial.

Responsible researcher: Gabriel Antonio Gazziero Moraca.
Institute of Biosciences, São Paulo State University, Rio Claro.
Adress: Av. 24-A, n° 1515, Bela Vista, CEP: 13506-900, Rio Claro/SP
Contac details: telephone (19) 98207-4760 e-mail: gabriel.moraca@unesp.br

Supervisor: Profa. Dra. Lilian Teresa Bucken Gobbi.
Institute of Biosciences, São Paulo State University, Rio Claro.
Adress: Av. 24-A, n 1515, Bela Vista, Rio Claro/SP.
Contact details: telephone (19) 3526-4365 e-mail: lilian.gobbi@unesp.br

Data about the Research participant:

Name:____

ID:____

Sex:_________________________________ Date of birth:_____/_____/_________________________

Adress:____

Contacts:____

CEP-IB/UNESP-CRC
Av. 24A, nº 1515 – Bela Vista – 13506-900 – Rio Claro/SP
Telephone: (19) 3526-9678
E-mail: cepib.rc@unesp.br

Number: ______________________________

______________________________ ______________________________
Participant rubric Researcher rubric
