# Supplementary material for: Aerobic exercise on the treadmill combined with transcranial direct current stimulation on the gait of people with Parkinson’s disease: A protocol for a randomized clinical trial
Source: PLoS One. 2024 Apr 25;19(4):e0300243. doi: 10.1371/journal.pone.0300243 (PMC11045059; doi:10.1371/journal.pone.0300243)
Supplement: S4 File — (PDF) [file pone.0300243.s005.pdf]

TERMO DE CONSENTIMENTO LIVRE E ESCLARECIDO
(Conselho Nacional de Saúde, Resolução 466/12)

Eu, Gabriel Antonio Gazziero Moraca, RG: 56.851.201-7, aluno de mestrado do Programa de Pós-Graduação em Ciências do Movimento – Interunidades e pesquisador responsável pelo estudo, convido o Senhor (a) a participar, como voluntário(a), da pesquisa intitulada "Efeitos da estimulação transcraniana por corrente contínua combinada com treino em esteira no andar de pacientes com doença de Parkinson: um ensaio clínico randomizado". Os membros da equipe de pesquisa são: a Profa. Dra. Lilian Teresa Bucken Gobbi, o Prof. Dr. Diego Orcioli-Silva, o Prof. Me. Victor Spiandor Beretta e a graduanda Beatriz Regina Legutke. Todos os procedimentos experimentais da pesquisa serão realizados no Laboratório de Estudos da Postura e da Locomoção (LEPLO), no Departamento de Educação Física do Instituto de Biociências da Universidade Estadual Paulista campus de Rio Claro (UNESP/RC).

O objetivo geral da pesquisa é verificar os efeitos agudos da estimulação transcraniana por corrente contínua (ETCC) anódica aplicada no córtex motor primário e no córtex pré-frontal combinada com treino em esteira no andar de pacientes com doença de Parkinson. O objetivo secundário é analisar a influência da combinação ETCC com treino em esteira na atividade do córtex pré-frontal em diferentes condições do andar, como o andar usual, com ultrapassagem de obstáculos e com tarefa dupla cognitiva.

Caso aceite participar desta pesquisa, o Senhor (a) será convidado (a) a comparecer no LEPLO em cinco dias, com intervalo de uma semana entre os quatro últimos dias. No dia 1, o Senhor (a) responderá questionários específicos sobre problemas de saúde, medicamentos em uso, estado cognitivo, grau de acometimento da doença de Parkinson, medo de quedas e congelamento do andar. Caso o Senhor (a) deseje, o seu acompanhante poderá estar junto durante as avaliações. Ainda no dia 1, serão coletados altura e peso, e o Senhor (a) será convidado a andar em uma esteira ergométrica para verificarmos a velocidade da esteira em que o Senhor (a) se sente confortável. Nos dias 2, 3, 4 e 5 o Senhor (a) participará das avaliações do andar e atividade cortical e do treino na esteira combinado com a ETCC. Todo o protocolo (avaliações e treino) deverá durar em torno de 1h10min por dia. Para facilitar o entendimento do Senhor (a) sobre os procedimentos que serão realizados, as avaliações e o treino estão descritos a seguir:

Rubrica do Participante ______________________ Rubrica do Pesquisador ______________________

Avaliações do andar e da atividade cortical: um avaliador treinado fará marcações na cabeça do Senhor (a) para colocar uma touca que irá registrar a atividade cortical. O contato da touca é apenas superficial e não causa dor ou desconforto. O avaliador colocará um acelerômetro nas costas do Senhor (a) que servirá para avaliar seu andar. Após estas preparações, o Senhor (a) andará, em sua velocidade habitual, em um circuito retangular em três condições: andar usual, andar com ultrapassagem de obstáculos e andar com tarefa dupla cognitiva. O Senhor (a) fará 3 tentativas em cada condição e cada tentativa terá duração total de 1 minuto. Durante os primeiros 30s, o Senhor (a) deverá ficar em pé parado, olhar para frente, em silêncio e realizar contagens simples mentalmente (1 + 1 + 1...). Quando o avaliador falar "prepara, vai", o Senhor (a) deverá andar por 30s no circuito. Na condição com ultrapassagem de obstáculos, quatro obstáculos com 15cm de altura serão colocados no circuito. Na condição com tarefa dupla cognitiva, um áudio tocará números de 1 a 9 e o Senhor (a) deverá contar, mentalmente, quantas vezes escutou determinados números. No final da tentativa, o Senhor (a) deverá falar a sua resposta. Todos estes procedimentos acontecerão antes e depois do treino na esteira combinada com a ETCC. Ao término das avaliações pré-treino, o avaliador irá retirar os equipamentos do Senhor (a), mas as marcações serão mantidas para garantir o mesmo posicionamento dos equipamentos nas avaliações pós-treino.

Treino na esteira combinada com a ETCC: antes de iniciar o treino, o Senhor (a) ficará sentado para aferição de sua pressão arterial e frequência cardíaca. Em seguida, o avaliador irá posicionar três eletrodos de esponja úmidos, fixados por meio de faixas elásticas, na cabeça do Senhor (a) para aplicação da estimulação. A estimulação não oferece riscos ao Senhor (a) devido à baixa intensidade da corrente elétrica, mas pode ocorrer ligeira sensação de formigamento e/ou coceira na área estimulada durante os primeiros segundos da aplicação. A estimulação terá duração total de 20 minutos e será realizada enquanto o Senhor (a) anda na esteira. O protocolo do treino na esteira terá duração total de 30 minutos, sendo que haverá: aquecimento (5 minutos e sem estimulação); parte principal (20 minutos e com estimulação) e volta a calma (5 minutos e sem estimulação). O avaliador irá controlar a velocidade da esteira e irá monitorar sua frequência cardíaca durante todo o treino. Por fim, o Senhor (a) deverá responder um questionário sobre as sensações causadas pela estimulação e sua pressão arterial será mensurada novamente.

Apesar dos procedimentos possuírem alto grau de segurança, há riscos de quedas, constrangimentos e desconfortos durante sua participação na pesquisa. Para minimizar estes riscos, todas as avaliações e os treinos serão aplicadas por profissionais experientes e treinados. Nas avaliações do andar e da atividade cortical (pré e pós-treino) haverá um avaliador ao seu lado para auxiliá-lo em caso de qualquer evento inesperado. Os obstáculos que serão utilizados são de espuma e em cores contrastantes, o que diminui os riscos de acidentes e facilita a identificação. Durante o treino na esteira com a ETCC, o Senhor (a) poderá sentir formigamento, queimação ou irritabilidade na região estimulada. Estes efeitos podem aparecer no início da estimulação, desaparecendo após alguns segundos. Um membro da equipe pedirá para o Senhor (a) informar qualquer sensação de desconforto de forma duradoura para que medidas de segurança sejam tomadas. Além disso, durante todo o treino, o Senhor (a) estará com um equipamento de segurança (arnês) preso no teto para evitar a ocorrência de quedas. Quando necessário, procedimentos de primeiros socorros serão prestados pelos membros da equipe e o Senhor (a) será encaminhado para o centro de saúde mais próximo.

Não haverá benefício imediato para sua saúde ao participar desta pesquisa, porém, os resultados desta pesquisa poderão servir para diversos propósitos. É possível que a pesquisa gere melhor entendimento sobre os efeitos de uma técnica de estimulação não invasiva combinada com exercício físico no andar, em diferentes situações, e na atividade cortical dos pacientes com doença de Parkinson. Ainda, esta pesquisa pode contribuir para o desenvolvimento de novos procedimentos de reabilitação que possam amenizar os comprometimentos motores que essa população possui.

O Senhor (a) receberá um código de identificação para manter o seu anonimato durante a pesquisa e poderá pedir esclarecimentos sobre os procedimentos a qualquer momento. A participação na pesquisa é voluntária, portanto, o Senhor (a) poderá se recusar a participar da pesquisa e/ou abandoná-la a qualquer momento, sem nenhum prejuízo financeiro ou pessoal. Ainda, ressalto que o Senhor (a) não receberá nenhum tipo de remuneração ao participar da pesquisa e não haverá despesas da sua parte. Todos os resultados do estudo serão usados, única e exclusivamente, para fins de ensino e pesquisa. O Senhor (a) poderá solicitar, a qualquer momento, informações sobre os resultados dos testes realizados e poderá entrar em contato com o Comitê de Ética em Pesquisa do Instituto de Biociências da UNESP/RC para quaisquer esclarecimentos sobre a pesquisa.

_______________________ Rubrica do Participante

_______________________ Rubrica do Pesquisador

Os seus dados pessoais, aqueles que possam identificá-lo (nome, RG, telefone, endereço e data de nascimento) nunca serão compartilhados e serão mantidos em sigilo absoluto. Convido-o (a) a responder as perguntas abaixo sobre o reuso e compartilhamento dos seus dados não identificadores (que não permitem detectar a sua identidade). Ressalto que o Senhor (a) pode retirar o consentimento sobre reuso e compartilhamento dos dados não identificadores a qualquer momento.

O Senhor (a) permite que os seus dados não identificadores coletados sejam armazenados e preservados em repositórios de dados, como o Repositório Institucional da UNESP?

☐ Sim ☐ Não

O Senhor (a) permite que os seus dados não identificadores coletados sejam publicados em revistas científicas específicas sobre dados de pesquisas?

☐ Sim ☐ Não

O Senhor (a) permite que os seus dados não identificadores coletados sejam utilizados em outros estudos por pesquisadores do LEPLO?

☐ Sim ☐ Não

O Senhor (a) permite que os seus dados não identificadores coletados sejam utilizados em outros estudos por pesquisadores de outras instituições?

☐ Sim ☐ Não

Se o Senhor (a) se sentir suficientemente esclarecido sobre esta pesquisa, seus objetivos, eventuais riscos e benefícios, convido-o (a) a assinar este Termo, elaborado em duas vias, sendo que uma ficará com o Senhor (a) e outra com o pesquisador responsável.

Rio Claro, ______ de _________________ de __________

_______________________________ _______________________________

Assinatura do participante da pesquisa Assinatura do pesquisador

Dados sobre a Pesquisa:

Título do Projeto: Efeitos da estimulação transcraniana por corrente contínua combinada com treino em esteira no andar de pacientes com doença de Parkinson: um ensaio clínico randomizado.

Pesquisador Responsável: Gabriel Antonio Gazziero Moraca.
Cargo/função: estudante de mestrado em Ciências do Movimento.
Instituição: Universidade Estadual Paulista, Instituto de Biociências, Rio Claro.
Endereço: Av. 24-A, n° 1515, Bela Vista, CEP: 13506-900, Rio Claro/SP
Dados para contato: fone (19) 98207-4760 e-mail: gabriel.moraca@unesp.br

Orientadora: Profa. Dra. Lilian Teresa Bucken Gobbi.
Cargo/função: Professora Titular.
Instituição: Universidade Estadual Paulista, Instituto de Biociências, Rio Claro.
Endereço: Av. 24-A, n 1515, Bela Vista, Rio Claro/SP.
Dados para contato: fone (19) 3526-4365 e-mail: lilian.gobbi@unesp.br

Dados sobre o participante da Pesquisa:

Nome:____

Documento de Identidade:____

Sexo:_______________________________ Data de Nascimento:_____/_____/________________

Endereço:____

Telefone para contato:____

CEP-IB/UNESP-CRC
Av. 24A, nº 1515 – Bela Vista – 13506-900 – Rio Claro/SP
Telefone: (19) 3526-9678
E-mail: cepib.rc@unesp.br

Número do parecer: ___________________________

Rubrica do Participante

Rubrica do Pesquisador
