# Supplementary material for: Aerobic exercise on the treadmill combined with transcranial direct current stimulation on the gait of people with Parkinson’s disease: A protocol for a randomized clinical trial
Source: PLoS One. 2024 Apr 25;19(4):e0300243. doi: 10.1371/journal.pone.0300243 (PMC11045059; doi:10.1371/journal.pone.0300243)
Supplement: Peer review history

# Editor report: Decision Letter 0

**DOI:** 10.1371/journal.pone.0300243.r001

**Authors:** Vanessa Carels

25 Feb 2024

Aerobic exercise on the treadmill combined with transcranial direct current stimulation on the gait of people with Parkinson's disease: A protocol for a randomized clinical trial.

PONE-D-24-01940

Dear Dr. Barbieri,

We’re pleased to inform you that your manuscript has been judged scientifically suitable for publication and will be formally accepted for publication once it meets all outstanding technical requirements.

Within one week, you’ll receive an e-mail detailing the required amendments. When these have been addressed, you’ll receive a formal acceptance letter and your manuscript will be scheduled for publication.

An invoice for payment will follow shortly after the formal acceptance. To ensure an efficient process, please log into Editorial Manager at http://www.editorialmanager.com/pone/, click the 'Update My Information' link at the top of the page, and double check that your user information is up-to-date. If you have any billing related questions, please contact our Author Billing department directly at authorbilling@plos.org.

If your institution or institutions have a press office, please notify them about your upcoming paper to help maximize its impact. If they’ll be preparing press materials, please inform our press team as soon as possible -- no later than 48 hours after receiving the formal acceptance. Your manuscript will remain under strict press embargo until 2 pm Eastern Time on the date of publication. For more information, please contact onepress@plos.org.

Kind regards,

Vanessa Carels

Staff Editor

PLOS ONE

Journal requirements:

When submitting your revision, we need you to address these additional requirements.

1. Please ensure that your manuscript meets PLOS ONE's style requirements, including those for file naming. The PLOS ONE style templates can be found at 

https://journals.plos.org/plosone/s/file?id=wjVg/PLOSOne_formatting_sample_main_body.pdf and 

https://journals.plos.org/plosone/s/file?id=ba62/PLOSOne_formatting_sample_title_authors_affiliations.pdf

2. Thank you for stating the following financial disclosure: "This study was supported by grants from the Brazilian Federal Agency for Support and Evaluation of Graduate Education (CAPES; grant number #88887.658629/2021-00 [GAGM]; #88887.598229/2021-00 [VCZ]); the Pró-Reitoria de Pesquisa (PROPe) of São Paulo State University (UNESP) [DOS]; [TMS], the São Paulo Research Foundation (FAPESP, grant number 2020/12656-1 [BRL]; 2023/02950-8 [PPG]), and the National Council for Scientific and Technological Development (CNPq; grant number #429549/2018-0; #309045/2017-7 [LTBG])." 

Please state what role the funders took in the study.  If the funders had no role, please state: ""The funders had no role in study design, data collection and analysis, decision to publish, or preparation of the manuscript."" 

If this statement is not correct you must amend it as needed. 

Please respond by return e-mail so that we can amend your financial disclosure and competing interests on your behalf.
